# Pseudo current density maps of electrophysiological heart, nerve or brain function and their physical basis

**DOI:** 10.1186/1477-044X-4-5

**Published:** 2006-10-13

**Authors:** Wolfgang Haberkorn, Uwe Steinhoff, Martin Burghoff, Olaf Kosch, Andreas Morguet, Hans Koch

**Affiliations:** 1 Physikalisch-Technische Bundesanstalt, Berlin, Germany; 2 Charité Campus Benjamin Franklin, Clinic II, Berlin, Germany

## Abstract

**Background:**

In recent years the visualization of biomagnetic measurement data by so-called pseudo current density maps or Hosaka-Cohen (HC) transformations became popular.

**Methods:**

The physical basis of these intuitive maps is clarified by means of analytically solvable problems.

**Results:**

Examples in magnetocardiography, magnetoencephalography and magnetoneurography demonstrate the usefulness of this method.

**Conclusion:**

Hardware realizations of the HC-transformation and some similar transformations are discussed which could advantageously support cross-platform comparability of biomagnetic measurements.

## Background

In 1976 Cohen et al. introduced in a sequence of publications a method to construct so-called pseudo current density- or arrow-maps from multichannel biomagnetic signals obtained by magnetocardiography (MCG) [1-4]. The purpose was to transform the measured magnetic field values in a way that the resulting maps could be more easily related to the underlying current density distribution. Later this method was frequently referred to as the Hosaka-Cohen (HC) transformation and its performance was analyzed in some detail [5,6]. However, it did not find widespread application until recent years, when a kind of renaissance of this method occurred. Recently, the HC-transformation is used in MCG [7-21], fetal MCG [22-24], magnetoencephalography (MEG) [25-27] and magnetoneurography (MNG) [28].

A reason for this new development may be the advance of computing power and visualization tools. In addition, in former times system designers preferred to display magnetic field maps (MFM), since they were interested in the measured physical quantity. However, for the end-user -the physicians- MFMs are not very instructive, as the MFM maximum values do not occur above those positions where the generating currents are flowing.

**Figure 1:**
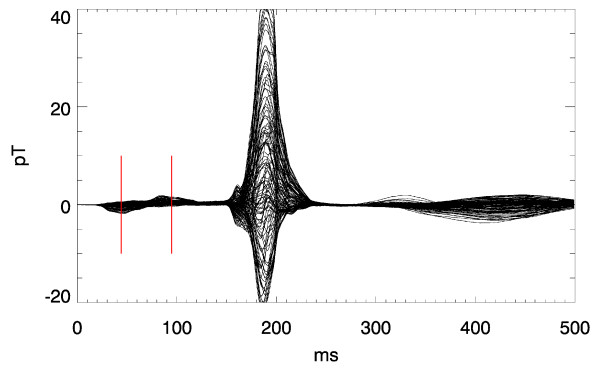
Butterfly plot of a multichannel magnetocardiogramm. The two cursors mark the time instants related to the visualizations shown in Fig. 2 and 3 respectively.

**Figure 2:**
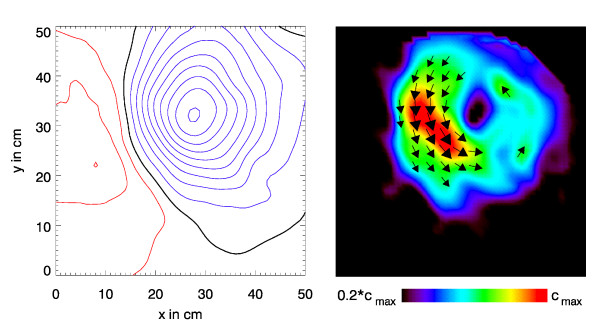
Visualization of the atrial activation for the first time instant marked by a cursor in Fig.1. Left: *B*_*z*_-map drawn as isocontour maps with a magnetic flux density difference of 0.5pT between adjacent contour lines (red: positive, i.e. directed towards the subject; blue: negative; black: *B*_*z *_= 0). Right: the corresponding pseudo current density map.

**Figure 3:**
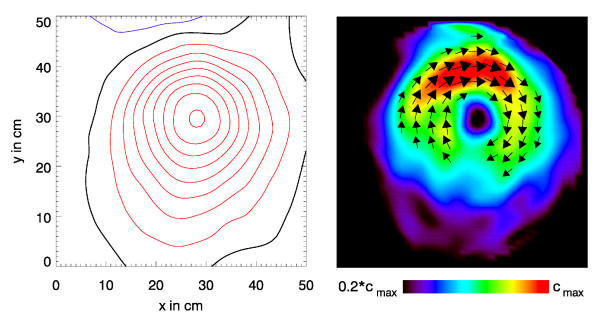
Visualization of the atrial activation for the second time instant marked by the respective cursor in Fig.1.

Figs. 1, 2, 3 illustrate this point: it shows two instants of the atrial excitation marked by the cursors in the MCG-butterfly-plot in Fig. 1 (a butterfly-plot is obtained by superpositioning the MCG-Signals of all channels in one display). The respective pseudo current density (PCD-) plots show very clearly and intuitively the preceding activation over the right atrium (Fig. 2, right) followed by that over the left atrium (Fig. 3, right), whereas the MFMs in Fig. 2 (left) and in Fig. 3 (left) require expert knowledge to interpret them in the same way.

Other features of modern pseudo current density maps helped to spur interest:

i) while Hosaka and Cohen coded the information of the pseudo current density amplitude into the size of the arrows the recent display techniques added an underlying false-colour scaling to the maps.

ii) visually attractive results are achieved, if a sequence of maps is presented as an animated clip. Then the spatio-temporal dynamics of the electrophysiological function are more easily perceptible.

The question that remains open is: what do pseudo current density maps really show? Already the term "pseudo" indicates that the real current density distribution is different and may deviate considerably. This is already evident when considering the fact that the PCD-maps are only 2D-projections of a 3D reality. The initial papers of Hosaka and Cohen just gave an empirical explanation, why their maps produce an approximate image of the underlying current density distribution. Later explanations e.g. by other authors [7] relating the curl of the measured magnetic induction curl B→
 MathType@MTEF@5@5@+=feaafiart1ev1aaatCvAUfKttLearuWrP9MDH5MBPbIqV92AaeXatLxBI9gBamXvP5wqSXMqHnxAJn0BKvguHDwzZbqegyvzYrwyUfgarqqtubsr4rNCHbGeaGqiA8vkIkVAFgIELiFeLkFeLk=iY=Hhbbf9v8qqaqFr0xc9pk0xbba9q8WqFfeaY=biLkVcLq=JHqVepeea0=as0db9vqpepesP0xe9Fve9Fve9GapdbaqaaeGacaGaaiaabeqaamqadiabaaGcbaGafmOqaiKbaSaaaaa@3E0A@ with the current density j→
 MathType@MTEF@5@5@+=feaafiart1ev1aaatCvAUfKttLearuWrP9MDH5MBPbIqV92AaeXatLxBI9gBaebbnrfifHhDYfgasaacH8akY=wiFfYdH8Gipec8Eeeu0xXdbba9frFj0=OqFfea0dXdd9vqai=hGuQ8kuc9pgc9s8qqaq=dirpe0xb9q8qiLsFr0=vr0=vr0dc8meaabaqaciaacaGaaeqabaqabeGadaaakeaacuWGQbGAgaWcaaaa@2E1B@ were incorrect and misleading. Therefore in the following chapters an analytically based calculation is presented that illustrates the physical justification and the limitations of this visualization method.

This paper will not deal with minimum norm estimates or other inverse methods calculating the current density from field maps. Rather, the Hosaka-Cohen transformation provides just another representation of the measured magnetic field by a postprocessing of the magnetic field data. The underlying current distribution does not enter in the calculation of the HC-transformation. We intend to clarify in which way certain features of the PCD-maps can nevertheless be related to the underlying current distribution. Some common fallacies in the interpretation of PCD maps are elucidated.

Finally we would like to stress the utility of PCD maps to provide a visualization of measurement results obtained by biomagnetic systems with very different sensor configurations. Whether magnetometers, planar gradiometers or vector magnetometers are used always similar PCD maps may be computed and will thus allow a simple cross-platform, i.e. multicentre comparability of biomagnetic investigations.

## Methods

### Construction of pseudo current density maps

PCD-maps are obtained from magnetic field values at a number of points in space [29,30]. Multichannel measurement systems, containing a number of SQUIDs (superconducting quantum interference devices) as magnetic field sensors, are used to measure the magnetic fields generated by electrophysiological functions in the heart (MCG = magnetocardiography), the brain (MEG = magnetoencephalography) or in other muscles or nerves (MNG = magnetoneurography). In MEG, helmet systems are used, where the SQUIDs are arranged on the surface of a sphere. For other applications, the SQUIDs are distributed more or less in a plane.

For the following discussion a *simple *current dipole source with current dipole moment

p→=Is→     (1)
 MathType@MTEF@5@5@+=feaafiart1ev1aaatCvAUfKttLearuWrP9MDH5MBPbIqV92AaeXatLxBI9gBaebbnrfifHhDYfgasaacH8akY=wiFfYdH8Gipec8Eeeu0xXdbba9frFj0=OqFfea0dXdd9vqai=hGuQ8kuc9pgc9s8qqaq=dirpe0xb9q8qiLsFr0=vr0=vr0dc8meaabaqaciaacaGaaeqabaqabeGadaaakeaacuWGWbaCgaWcaiabg2da9iabdMeajjqbdohaZzaalaGaaCzcaiaaxMaadaqadaqaaiabigdaXaGaayjkaiaawMcaaaaa@3586@

may be considered, i.e. a source-drain configuration with vanishing source-drain distance with unit vector s→
 MathType@MTEF@5@5@+=feaafiart1ev1aaatCvAUfKttLearuWrP9MDH5MBPbIqV92AaeXatLxBI9gBaebbnrfifHhDYfgasaacH8akY=wiFfYdH8Gipec8Eeeu0xXdbba9frFj0=OqFfea0dXdd9vqai=hGuQ8kuc9pgc9s8qqaq=dirpe0xb9q8qiLsFr0=vr0=vr0dc8meaabaqaciaacaGaaeqabaqabeGadaaakeaacuWGZbWCgaWcaaaa@2E2D@ and a source strength of *I*. This current element generates a magnetic flux density B→
 MathType@MTEF@5@5@+=feaafiart1ev1aaatCvAUfKttLearuWrP9MDH5MBPbIqV92AaeXatLxBI9gBamXvP5wqSXMqHnxAJn0BKvguHDwzZbqegyvzYrwyUfgarqqtubsr4rNCHbGeaGqiA8vkIkVAFgIELiFeLkFeLk=iY=Hhbbf9v8qqaqFr0xc9pk0xbba9q8WqFfeaY=biLkVcLq=JHqVepeea0=as0db9vqpepesP0xe9Fve9Fve9GapdbaqaaeGacaGaaiaabeqaamqadiabaaGcbaGafmOqaiKbaSaaaaa@3E0A@(r→
 MathType@MTEF@5@5@+=feaafiart1ev1aaatCvAUfKttLearuWrP9MDH5MBPbIqV92AaeXatLxBI9gBamXvP5wqSXMqHnxAJn0BKvguHDwzZbqegyvzYrwyUfgarqqtubsr4rNCHbGeaGqiA8vkIkVAFgIELiFeLkFeLk=iY=Hhbbf9v8qqaqFr0xc9pk0xbba9q8WqFfeaY=biLkVcLq=JHqVepeea0=as0db9vqpepesP0xe9Fve9Fve9GapdbaqaaeGacaGaaiaabeqaamqadiabaaGcbaGafmOCaiNbaSaaaaa@3E6A@) which – according to the law of Biot-Savart – is expressed as

B→(r→)=μ04πp→×r→r3.     (2)
 MathType@MTEF@5@5@+=feaafiart1ev1aaatCvAUfKttLearuWrP9MDH5MBPbIqV92AaeXatLxBI9gBaebbnrfifHhDYfgasaacH8akY=wiFfYdH8Gipec8Eeeu0xXdbba9frFj0=OqFfea0dXdd9vqai=hGuQ8kuc9pgc9s8qqaq=dirpe0xb9q8qiLsFr0=vr0=vr0dc8meaabaqaciaacaGaaeqabaqabeGadaaakeaacuWGcbGqgaWcaiabcIcaOiqbdkhaYzaalaGaeiykaKIaeyypa0ZaaSaaaeaaiiGacqWF8oqBdaWgaaWcbaGaeGimaadabeaaaOqaaiabisda0iab=b8aWbaadaWcaaqaaiqbdchaWzaalaGaey41aqRafmOCaiNbaSaaaeaacqWGYbGCdaahaaWcbeqaaiabiodaZaaaaaGccqGGUaGlcaWLjaGaaCzcamaabmaabaGaeGOmaidacaGLOaGaayzkaaaaaa@43FD@

**Figure 4:**
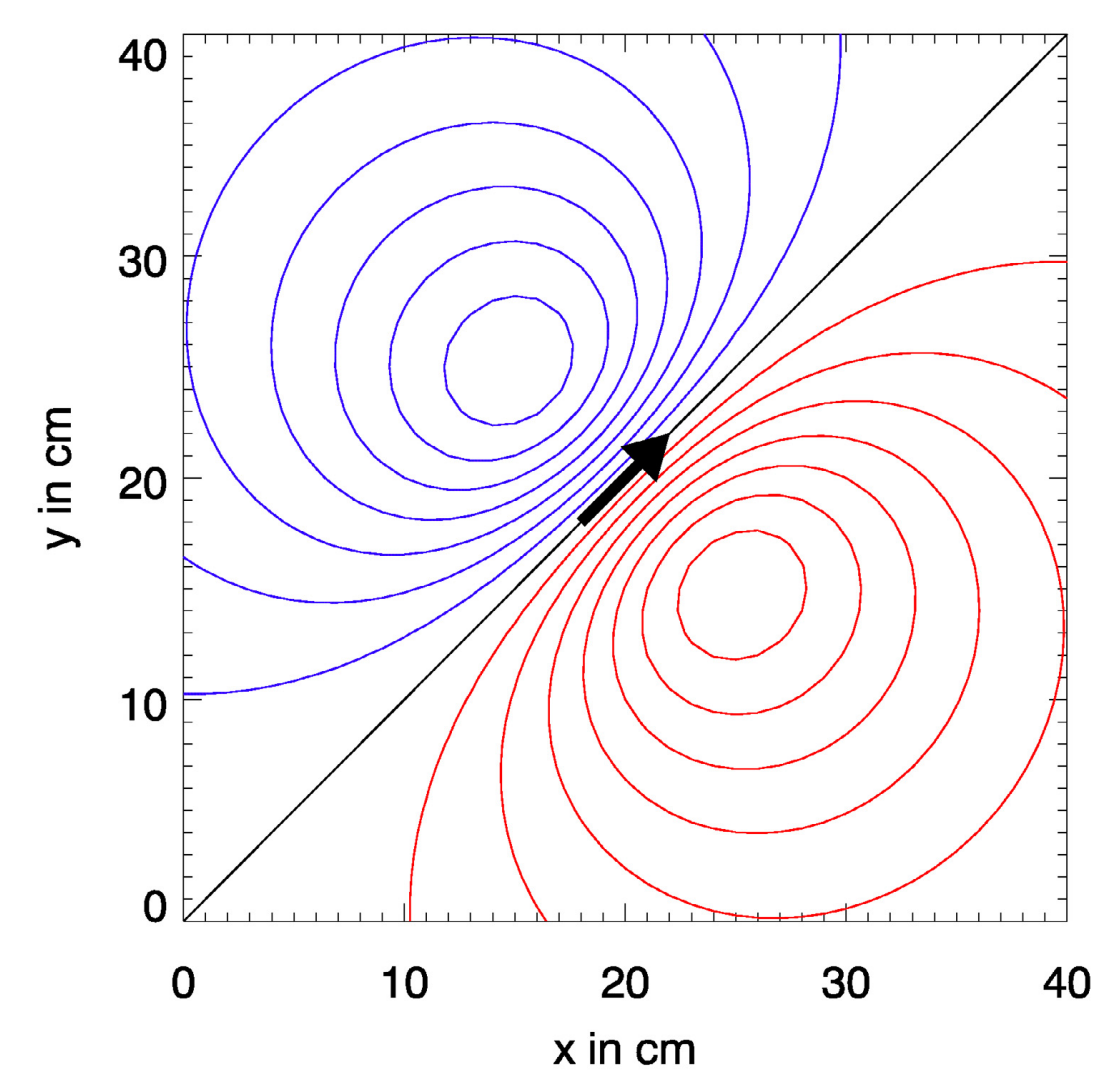
*B*_*z*_-map of the magnetic flux density calculated from Biot-Savart's law for a current dipole (|p→
 MathType@MTEF@5@5@+=feaafiart1ev1aaatCvAUfKttLearuWrP9MDH5MBPbIqV92AaeXatLxBI9gBaebbnrfifHhDYfgasaacH8akY=wiFfYdH8Gipec8Eeeu0xXdbba9frFj0=OqFfea0dXdd9vqai=hGuQ8kuc9pgc9s8qqaq=dirpe0xb9q8qiLsFr0=vr0=vr0dc8meaabaqaciaacaGaaeqabaqabeGadaaakeaacuWGWbaCgaWcaaaa@2E27@| = 1 μAm) at a position 10 cm below the map's plane with a *x*-*y*-projection as indicated by the arrow.

**Figure 5:**
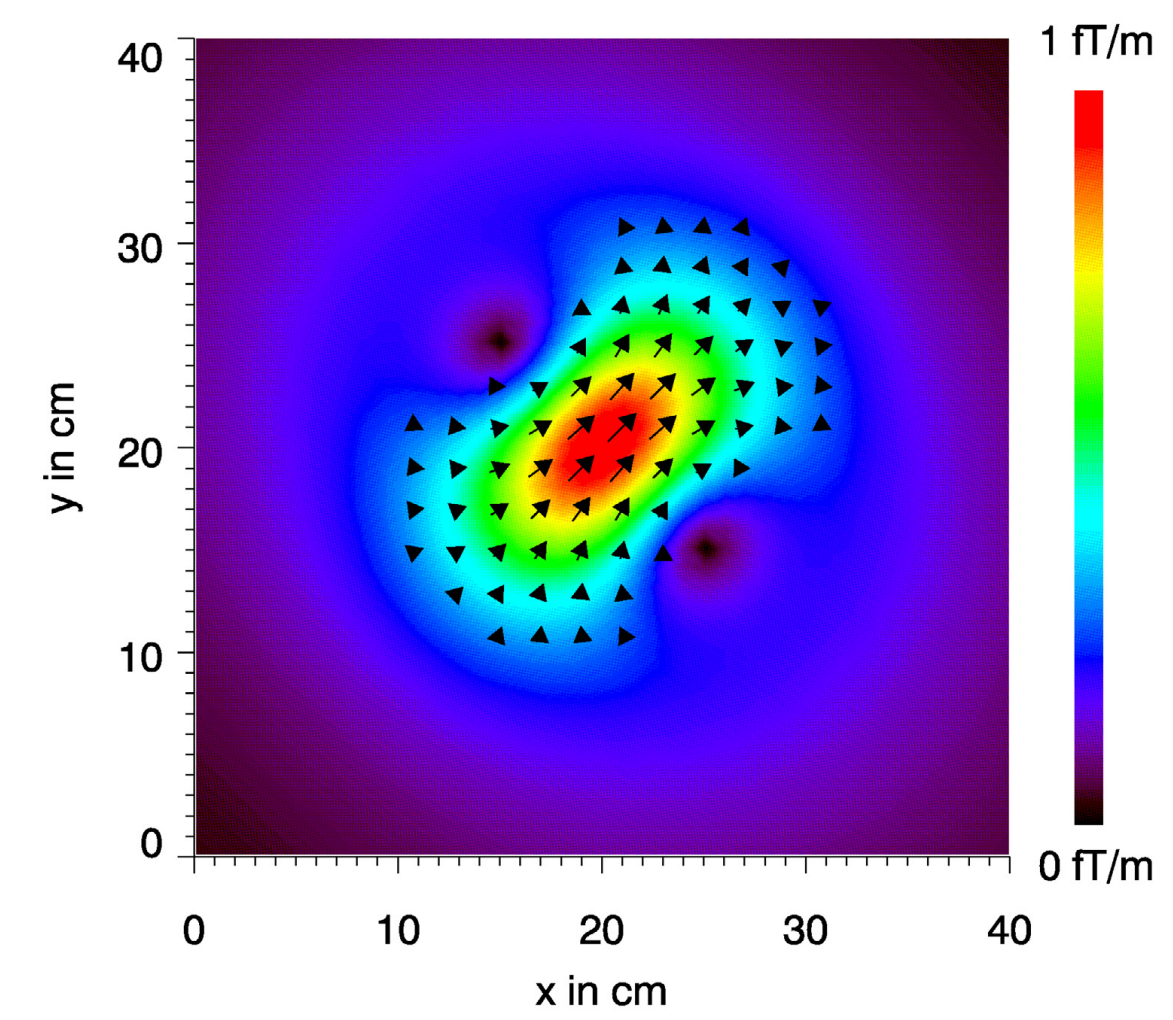
Psydo current density map corresponding to Fig. 4.

Often, planar SQUID-systems measure only one component of the B→
 MathType@MTEF@5@5@+=feaafiart1ev1aaatCvAUfKttLearuWrP9MDH5MBPbIqV92AaeXatLxBI9gBamXvP5wqSXMqHnxAJn0BKvguHDwzZbqegyvzYrwyUfgarqqtubsr4rNCHbGeaGqiA8vkIkVAFgIELiFeLkFeLk=iY=Hhbbf9v8qqaqFr0xc9pk0xbba9q8WqFfeaY=biLkVcLq=JHqVepeea0=as0db9vqpepesP0xe9Fve9Fve9GapdbaqaaeGacaGaaiaabeqaamqadiabaaGcbaGafmOqaiKbaSaaaaa@3E0A@-field, e.g.*B*_*z*_. Fig. 4 shows the *B*_*z*_-distribution calculated with (2) for a measurement plane of size 40 cm × 40 cm which is positioned 10 cm above a current dipole with a dipole moment of 1 μAm. The direction of the dipole within the *x*-*y*-plane is diagonal to the coordinate system and the magnetic flux density distribution is presented as an isocontour plot. The difference between two adjacent contour lines corresponds to a magnetic flux density difference of 0.5 pT. Red lines correspond to positive *B*_*z*_-values and blue lines to negative *B*_*z*_-values and the black line marks *B*_*z *_= 0. The pseudo current density c→
 MathType@MTEF@5@5@+=feaafiart1ev1aaatCvAUfKttLearuWrP9MDH5MBPbIqV92AaeXatLxBI9gBaebbnrfifHhDYfgasaacH8akY=wiFfYdH8Gipec8Eeeu0xXdbba9frFj0=OqFfea0dXdd9vqai=hGuQ8kuc9pgc9s8qqaq=dirpe0xb9q8qiLsFr0=vr0=vr0dc8meaabaqaciaacaGaaeqabaqabeGadaaakeaacuWGJbWygaWcaaaa@2E0D@(*x*, *y*) in the map in Fig. 5 is gained from the *B*_*z*_-values by the following transformation

c→=∂Bz∂ye→x−∂Bz∂xe→y.     (3)
 MathType@MTEF@5@5@+=feaafiart1ev1aaatCvAUfKttLearuWrP9MDH5MBPbIqV92AaeXatLxBI9gBaebbnrfifHhDYfgasaacH8akY=wiFfYdH8Gipec8Eeeu0xXdbba9frFj0=OqFfea0dXdd9vqai=hGuQ8kuc9pgc9s8qqaq=dirpe0xb9q8qiLsFr0=vr0=vr0dc8meaabaqaciaacaGaaeqabaqabeGadaaakeaacuWGJbWygaWcaiabg2da9maalaaabaGaeyOaIyRaemOqai0aaSbaaSqaaiabdQha6bqabaaakeaacqGHciITcqWG5bqEaaGafmyzauMbaSaadaWgaaWcbaGaemiEaGhabeaakiabgkHiTmaalaaabaGaeyOaIyRaemOqai0aaSbaaSqaaiabdQha6bqabaaakeaacqGHciITcqWG4baEaaGafmyzauMbaSaadaWgaaWcbaGaemyEaKhabeaakiabc6caUiaaxMaacaWLjaWaaeWaaeaacqaIZaWmaiaawIcacaGLPaaaaaa@48FB@

Thus the slopes of the *B*_*z*_-surface function determine the amplitude and the direction of the pseudo current arrows c→
 MathType@MTEF@5@5@+=feaafiart1ev1aaatCvAUfKttLearuWrP9MDH5MBPbIqV92AaeXatLxBI9gBaebbnrfifHhDYfgasaacH8akY=wiFfYdH8Gipec8Eeeu0xXdbba9frFj0=OqFfea0dXdd9vqai=hGuQ8kuc9pgc9s8qqaq=dirpe0xb9q8qiLsFr0=vr0=vr0dc8meaabaqaciaacaGaaeqabaqabeGadaaakeaacuWGJbWygaWcaaaa@2E0D@(*x*, *y*). e→
 MathType@MTEF@5@5@+=feaafiart1ev1aaatCvAUfKttLearuWrP9MDH5MBPbIqV92AaeXatLxBI9gBaebbnrfifHhDYfgasaacH8akY=wiFfYdH8Gipec8Eeeu0xXdbba9frFj0=OqFfea0dXdd9vqai=hGuQ8kuc9pgc9s8qqaq=dirpe0xb9q8qiLsFr0=vr0=vr0dc8meaabaqaciaacaGaaeqabaqabeGadaaakeaacuWGLbqzgaWcaaaa@2E11@_*x *_and e→
 MathType@MTEF@5@5@+=feaafiart1ev1aaatCvAUfKttLearuWrP9MDH5MBPbIqV92AaeXatLxBI9gBaebbnrfifHhDYfgasaacH8akY=wiFfYdH8Gipec8Eeeu0xXdbba9frFj0=OqFfea0dXdd9vqai=hGuQ8kuc9pgc9s8qqaq=dirpe0xb9q8qiLsFr0=vr0=vr0dc8meaabaqaciaacaGaaeqabaqabeGadaaakeaacuWGLbqzgaWcaaaa@2E11@_*y *_are the unit vectors in *x*- and *y*-direction.

In practice the partial differential ratios ∂Bz∂x
 MathType@MTEF@5@5@+=feaafiart1ev1aaatCvAUfKttLearuWrP9MDH5MBPbIqV92AaeXatLxBI9gBaebbnrfifHhDYfgasaacH8akY=wiFfYdH8Gipec8Eeeu0xXdbba9frFj0=OqFfea0dXdd9vqai=hGuQ8kuc9pgc9s8qqaq=dirpe0xb9q8qiLsFr0=vr0=vr0dc8meaabaqaciaacaGaaeqabaqabeGadaaakeaadaWcaaqaaiabgkGi2kabdkeacnaaBaaaleaacqWG6bGEaeqaaaGcbaGaeyOaIyRaemiEaGhaaaaa@33C1@ and ∂Bz∂y
 MathType@MTEF@5@5@+=feaafiart1ev1aaatCvAUfKttLearuWrP9MDH5MBPbIqV92AaeXatLxBI9gBaebbnrfifHhDYfgasaacH8akY=wiFfYdH8Gipec8Eeeu0xXdbba9frFj0=OqFfea0dXdd9vqai=hGuQ8kuc9pgc9s8qqaq=dirpe0xb9q8qiLsFr0=vr0=vr0dc8meaabaqaciaacaGaaeqabaqabeGadaaakeaadaWcaaqaaiabgkGi2kabdkeacnaaBaaaleaacqWG6bGEaeqaaaGcbaGaeyOaIyRaemyEaKhaaaaa@33C3@ are approximated by the difference ratios ΔBzΔx
 MathType@MTEF@5@5@+=feaafiart1ev1aaatCvAUfKttLearuWrP9MDH5MBPbIqV92AaeXatLxBI9gBaebbnrfifHhDYfgasaacH8akY=wiFfYdH8Gipec8Eeeu0xXdbba9frFj0=OqFfea0dXdd9vqai=hGuQ8kuc9pgc9s8qqaq=dirpe0xb9q8qiLsFr0=vr0=vr0dc8meaabaqaciaacaGaaeqabaqabeGadaaakeaadaWcaaqaaiabfs5aejabdkeacnaaBaaaleaacqWG6bGEaeqaaaGcbaGaeuiLdqKaemiEaGhaaaaa@33C1@ and ΔBzΔy
 MathType@MTEF@5@5@+=feaafiart1ev1aaatCvAUfKttLearuWrP9MDH5MBPbIqV92AaeXatLxBI9gBaebbnrfifHhDYfgasaacH8akY=wiFfYdH8Gipec8Eeeu0xXdbba9frFj0=OqFfea0dXdd9vqai=hGuQ8kuc9pgc9s8qqaq=dirpe0xb9q8qiLsFr0=vr0=vr0dc8meaabaqaciaacaGaaeqabaqabeGadaaakeaadaWcaaqaaiabfs5aejabdkeacnaaBaaaleaacqWG6bGEaeqaaaGcbaGaeuiLdqKaemyEaKhaaaaa@33C3@. They in turn may be easily obtained by utilizing the smooth surface function Bz(xi,yj)|i=0,...,n|j=0,...,m
 MathType@MTEF@5@5@+=feaafiart1ev1aaatCvAUfKttLearuWrP9MDH5MBPbIqV92AaeXatLxBI9gBaebbnrfifHhDYfgasaacH8akY=wiFfYdH8Gipec8Eeeu0xXdbba9frFj0=OqFfea0dXdd9vqai=hGuQ8kuc9pgc9s8qqaq=dirpe0xb9q8qiLsFr0=vr0=vr0dc8meaabaqaciaacaGaaeqabaqabeGadaaakeaadaabcaqaamaaeiaabaGaemOqai0aaSbaaSqaaiabdQha6bqabaGcdaqadaqaaiabdIha4naaBaaaleaacqWGPbqAaeqaaOGaeiilaWIaemyEaK3aaSbaaSqaaiabdQgaQbqabaaakiaawIcacaGLPaaaaiaawIa7amaaBaaaleaacqWGPbqAcqGH9aqpcqaIWaamcqGGSaalcqGGUaGlcqGGUaGlcqGGUaGlcqGGSaalcqWGUbGBaeqaaaGccaGLiWoadaWgaaWcbaGaemOAaOMaeyypa0JaeGimaaJaeiilaWIaeiOla4IaeiOla4IaeiOla4IaeiilaWIaemyBa0gabeaaaaa@4DBB@ that has been used to construct the map in Fig. 4.

The arrows drawn in Fig. 5 represent the c→
 MathType@MTEF@5@5@+=feaafiart1ev1aaatCvAUfKttLearuWrP9MDH5MBPbIqV92AaeXatLxBI9gBaebbnrfifHhDYfgasaacH8akY=wiFfYdH8Gipec8Eeeu0xXdbba9frFj0=OqFfea0dXdd9vqai=hGuQ8kuc9pgc9s8qqaq=dirpe0xb9q8qiLsFr0=vr0=vr0dc8meaabaqaciaacaGaaeqabaqabeGadaaakeaacuWGJbWygaWcaaaa@2E0D@-vectors at the respective coordinates. However, only the strongest c→
 MathType@MTEF@5@5@+=feaafiart1ev1aaatCvAUfKttLearuWrP9MDH5MBPbIqV92AaeXatLxBI9gBaebbnrfifHhDYfgasaacH8akY=wiFfYdH8Gipec8Eeeu0xXdbba9frFj0=OqFfea0dXdd9vqai=hGuQ8kuc9pgc9s8qqaq=dirpe0xb9q8qiLsFr0=vr0=vr0dc8meaabaqaciaacaGaaeqabaqabeGadaaakeaacuWGJbWygaWcaaaa@2E0D@-vectors are drawn to obtain a clearer picture. Although the amplitude of c→
 MathType@MTEF@5@5@+=feaafiart1ev1aaatCvAUfKttLearuWrP9MDH5MBPbIqV92AaeXatLxBI9gBaebbnrfifHhDYfgasaacH8akY=wiFfYdH8Gipec8Eeeu0xXdbba9frFj0=OqFfea0dXdd9vqai=hGuQ8kuc9pgc9s8qqaq=dirpe0xb9q8qiLsFr0=vr0=vr0dc8meaabaqaciaacaGaaeqabaqabeGadaaakeaacuWGJbWygaWcaaaa@2E0D@ is coded as the arrow length, a map with just those arrows is not as intuitive as the image shown. By underlying a false-color map scaled by the amplitude |c→
 MathType@MTEF@5@5@+=feaafiart1ev1aaatCvAUfKttLearuWrP9MDH5MBPbIqV92AaeXatLxBI9gBaebbnrfifHhDYfgasaacH8akY=wiFfYdH8Gipec8Eeeu0xXdbba9frFj0=OqFfea0dXdd9vqai=hGuQ8kuc9pgc9s8qqaq=dirpe0xb9q8qiLsFr0=vr0=vr0dc8meaabaqaciaacaGaaeqabaqabeGadaaakeaacuWGJbWygaWcaaaa@2E0D@| a considerable visual enhancement of the information is achieved.

As to be anticipated the maximum amplitude occurs just above the source and also the directions of the central strongest arrowc→
 MathType@MTEF@5@5@+=feaafiart1ev1aaatCvAUfKttLearuWrP9MDH5MBPbIqV92AaeXatLxBI9gBaebbnrfifHhDYfgasaacH8akY=wiFfYdH8Gipec8Eeeu0xXdbba9frFj0=OqFfea0dXdd9vqai=hGuQ8kuc9pgc9s8qqaq=dirpe0xb9q8qiLsFr0=vr0=vr0dc8meaabaqaciaacaGaaeqabaqabeGadaaakeaacuWGJbWygaWcaaaa@2E0D@ and that of the current dipole p→
 MathType@MTEF@5@5@+=feaafiart1ev1aaatCvAUfKttLearuWrP9MDH5MBPbIqV92AaeXatLxBI9gBaebbnrfifHhDYfgasaacH8akY=wiFfYdH8Gipec8Eeeu0xXdbba9frFj0=OqFfea0dXdd9vqai=hGuQ8kuc9pgc9s8qqaq=dirpe0xb9q8qiLsFr0=vr0=vr0dc8meaabaqaciaacaGaaeqabaqabeGadaaakeaacuWGWbaCgaWcaaaa@2E27@ coincide. On the other hand the PCD-map does not reproduce the point-like character of the current dipole! It is rather a characteristic point-spread function of the source.

Another interesting point to mention: the Hosaka-Cohen transformation utilizes two terms of

curl B→=(∂Bz∂y−∂By∂z)e→x+(∂Bx∂z−∂Bz∂x)e→y+(∂By∂x−∂Bx∂y)e→z.     (4)
 MathType@MTEF@5@5@+=feaafiart1ev1aaatCvAUfKttLearuWrP9MDH5MBPbIqV92AaeXatLxBI9gBaebbnrfifHhDYfgasaacH8akY=wiFfYdH8Gipec8Eeeu0xXdbba9frFj0=OqFfea0dXdd9vqai=hGuQ8kuc9pgc9s8qqaq=dirpe0xb9q8qiLsFr0=vr0=vr0dc8meaabaqaciaacaGaaeqabaqabeGadaaakeaacqqGJbWycqqG1bqDcqqGYbGCcqqGSbaBcqqGGaaicuWGcbGqgaWcaiabg2da9maabmaabaWaaSaaaeaacqGHciITcqWGcbGqdaWgaaWcbaGaemOEaOhabeaaaOqaaiabgkGi2kabdMha5baacqGHsisldaWcaaqaaiabgkGi2kabdkeacnaaBaaaleaacqWG5bqEaeqaaaGcbaGaeyOaIyRaemOEaOhaaaGaayjkaiaawMcaaiqbdwgaLzaalaWaaSbaaSqaaiabdIha4bqabaGccqGHRaWkdaqadaqaamaalaaabaGaeyOaIyRaemOqai0aaSbaaSqaaiabdIha4bqabaaakeaacqGHciITcqWG6bGEaaGaeyOeI0YaaSaaaeaacqGHciITcqWGcbGqdaWgaaWcbaGaemOEaOhabeaaaOqaaiabgkGi2kabdIha4baaaiaawIcacaGLPaaacuWGLbqzgaWcamaaBaaaleaacqWG5bqEaeqaaOGaey4kaSYaaeWaaeaadaWcaaqaaiabgkGi2kabdkeacnaaBaaaleaacqWG5bqEaeqaaaGcbaGaeyOaIyRaemiEaGhaaiabgkHiTmaalaaabaGaeyOaIyRaemOqai0aaSbaaSqaaiabdIha4bqabaaakeaacqGHciITcqWG5bqEaaaacaGLOaGaayzkaaGafmyzauMbaSaadaWgaaWcbaGaemOEaOhabeaakiabc6caUiaaxMaacaWLjaWaaeWaaeaacqaI0aanaiaawIcacaGLPaaaaaa@76AD@

As – according to Maxwell – curl B→
 MathType@MTEF@5@5@+=feaafiart1ev1aaatCvAUfKttLearuWrP9MDH5MBPbIqV92AaeXatLxBI9gBamXvP5wqSXMqHnxAJn0BKvguHDwzZbqegyvzYrwyUfgarqqtubsr4rNCHbGeaGqiA8vkIkVAFgIELiFeLkFeLk=iY=Hhbbf9v8qqaqFr0xc9pk0xbba9q8WqFfeaY=biLkVcLq=JHqVepeea0=as0db9vqpepesP0xe9Fve9Fve9GapdbaqaaeGacaGaaiaabeqaamqadiabaaGcbaGafmOqaiKbaSaaaaa@3E0A@ = μ_0_j→
 MathType@MTEF@5@5@+=feaafiart1ev1aaatCvAUfKttLearuWrP9MDH5MBPbIqV92AaeXatLxBI9gBaebbnrfifHhDYfgasaacH8akY=wiFfYdH8Gipec8Eeeu0xXdbba9frFj0=OqFfea0dXdd9vqai=hGuQ8kuc9pgc9s8qqaq=dirpe0xb9q8qiLsFr0=vr0=vr0dc8meaabaqaciaacaGaaeqabaqabeGadaaakeaacuWGQbGAgaWcaaaa@2E1B@ some authors concluded that this is the rationale for the pseudo-current density maps. However, at the sites where B→
 MathType@MTEF@5@5@+=feaafiart1ev1aaatCvAUfKttLearuWrP9MDH5MBPbIqV92AaeXatLxBI9gBamXvP5wqSXMqHnxAJn0BKvguHDwzZbqegyvzYrwyUfgarqqtubsr4rNCHbGeaGqiA8vkIkVAFgIELiFeLkFeLk=iY=Hhbbf9v8qqaqFr0xc9pk0xbba9q8WqFfeaY=biLkVcLq=JHqVepeea0=as0db9vqpepesP0xe9Fve9Fve9GapdbaqaaeGacaGaaiaabeqaamqadiabaaGcbaGafmOqaiKbaSaaaaa@3E0A@ is measured the current density is zero and thus also curl B→
 MathType@MTEF@5@5@+=feaafiart1ev1aaatCvAUfKttLearuWrP9MDH5MBPbIqV92AaeXatLxBI9gBamXvP5wqSXMqHnxAJn0BKvguHDwzZbqegyvzYrwyUfgarqqtubsr4rNCHbGeaGqiA8vkIkVAFgIELiFeLkFeLk=iY=Hhbbf9v8qqaqFr0xc9pk0xbba9q8WqFfeaY=biLkVcLq=JHqVepeea0=as0db9vqpepesP0xe9Fve9Fve9GapdbaqaaeGacaGaaiaabeqaamqadiabaaGcbaGafmOqaiKbaSaaaaa@3E0A@ = 0 holds. Hence no direct relation between j→
 MathType@MTEF@5@5@+=feaafiart1ev1aaatCvAUfKttLearuWrP9MDH5MBPbIqV92AaeXatLxBI9gBaebbnrfifHhDYfgasaacH8akY=wiFfYdH8Gipec8Eeeu0xXdbba9frFj0=OqFfea0dXdd9vqai=hGuQ8kuc9pgc9s8qqaq=dirpe0xb9q8qiLsFr0=vr0=vr0dc8meaabaqaciaacaGaaeqabaqabeGadaaakeaacuWGQbGAgaWcaaaa@2E1B@ and c→
 MathType@MTEF@5@5@+=feaafiart1ev1aaatCvAUfKttLearuWrP9MDH5MBPbIqV92AaeXatLxBI9gBaebbnrfifHhDYfgasaacH8akY=wiFfYdH8Gipec8Eeeu0xXdbba9frFj0=OqFfea0dXdd9vqai=hGuQ8kuc9pgc9s8qqaq=dirpe0xb9q8qiLsFr0=vr0=vr0dc8meaabaqaciaacaGaaeqabaqabeGadaaakeaacuWGJbWygaWcaaaa@2E0D@ and curl B→
 MathType@MTEF@5@5@+=feaafiart1ev1aaatCvAUfKttLearuWrP9MDH5MBPbIqV92AaeXatLxBI9gBamXvP5wqSXMqHnxAJn0BKvguHDwzZbqegyvzYrwyUfgarqqtubsr4rNCHbGeaGqiA8vkIkVAFgIELiFeLkFeLk=iY=Hhbbf9v8qqaqFr0xc9pk0xbba9q8WqFfeaY=biLkVcLq=JHqVepeea0=as0db9vqpepesP0xe9Fve9Fve9GapdbaqaaeGacaGaaiaabeqaamqadiabaaGcbaGafmOqaiKbaSaaaaa@3E0A@ exists at the location of the sensors. But since curl B→
 MathType@MTEF@5@5@+=feaafiart1ev1aaatCvAUfKttLearuWrP9MDH5MBPbIqV92AaeXatLxBI9gBamXvP5wqSXMqHnxAJn0BKvguHDwzZbqegyvzYrwyUfgarqqtubsr4rNCHbGeaGqiA8vkIkVAFgIELiFeLkFeLk=iY=Hhbbf9v8qqaqFr0xc9pk0xbba9q8WqFfeaY=biLkVcLq=JHqVepeea0=as0db9vqpepesP0xe9Fve9Fve9GapdbaqaaeGacaGaaiaabeqaamqadiabaaGcbaGafmOqaiKbaSaaaaa@3E0A@ = 0 everywhere in the measurement space, the two terms that represents c→
 MathType@MTEF@5@5@+=feaafiart1ev1aaatCvAUfKttLearuWrP9MDH5MBPbIqV92AaeXatLxBI9gBaebbnrfifHhDYfgasaacH8akY=wiFfYdH8Gipec8Eeeu0xXdbba9frFj0=OqFfea0dXdd9vqai=hGuQ8kuc9pgc9s8qqaq=dirpe0xb9q8qiLsFr0=vr0=vr0dc8meaabaqaciaacaGaaeqabaqabeGadaaakeaacuWGJbWygaWcaaaa@2E0D@ must exactly compensate the remaining terms of curl B→
 MathType@MTEF@5@5@+=feaafiart1ev1aaatCvAUfKttLearuWrP9MDH5MBPbIqV92AaeXatLxBI9gBamXvP5wqSXMqHnxAJn0BKvguHDwzZbqegyvzYrwyUfgarqqtubsr4rNCHbGeaGqiA8vkIkVAFgIELiFeLkFeLk=iY=Hhbbf9v8qqaqFr0xc9pk0xbba9q8WqFfeaY=biLkVcLq=JHqVepeea0=as0db9vqpepesP0xe9Fve9Fve9GapdbaqaaeGacaGaaiaabeqaamqadiabaaGcbaGafmOqaiKbaSaaaaa@3E0A@ (from equation 4). This in turn leads to the conclusion that also the remaining terms lead to equivalent arrow maps, as it is discussed later in this paper.

## Results

### Pseudo current density maps of analytically solvable models

A single current dipole alone (i.e. without considering return currents) and the application of Biot-Savart's law describe a too artificial, non-physical situation. The physical background of the PCD-maps may be evaluated by:

i) modeling the MCG by a current dipole in a conductive half space, and

ii) modeling the MNG by an extended linear or curved source [28,31] or by a train of current dipoles in a conducting half space and

iii) modeling the MEG by a current dipole in a conductive sphere.

Of course also those are quite crude models of the reality but they represent basic models of sound physics and can be treated completely analytical. Thus the relations between source and PCD-map and the role of curl B→
 MathType@MTEF@5@5@+=feaafiart1ev1aaatCvAUfKttLearuWrP9MDH5MBPbIqV92AaeXatLxBI9gBamXvP5wqSXMqHnxAJn0BKvguHDwzZbqegyvzYrwyUfgarqqtubsr4rNCHbGeaGqiA8vkIkVAFgIELiFeLkFeLk=iY=Hhbbf9v8qqaqFr0xc9pk0xbba9q8WqFfeaY=biLkVcLq=JHqVepeea0=as0db9vqpepesP0xe9Fve9Fve9GapdbaqaaeGacaGaaiaabeqaamqadiabaaGcbaGafmOqaiKbaSaaaaa@3E0A@ are exactly traceable.

#### Current dipole in a conductive half space

To a first approximation the MCG may be modeled by a current dipole p→
 MathType@MTEF@5@5@+=feaafiart1ev1aaatCvAUfKttLearuWrP9MDH5MBPbIqV92AaeXatLxBI9gBaebbnrfifHhDYfgasaacH8akY=wiFfYdH8Gipec8Eeeu0xXdbba9frFj0=OqFfea0dXdd9vqai=hGuQ8kuc9pgc9s8qqaq=dirpe0xb9q8qiLsFr0=vr0=vr0dc8meaabaqaciaacaGaaeqabaqabeGadaaakeaacuWGWbaCgaWcaaaa@2E27@ = (*p*_*x*_, *p*_*y*_, *p*_*z*_) at r→
 MathType@MTEF@5@5@+=feaafiart1ev1aaatCvAUfKttLearuWrP9MDH5MBPbIqV92AaeXatLxBI9gBamXvP5wqSXMqHnxAJn0BKvguHDwzZbqegyvzYrwyUfgarqqtubsr4rNCHbGeaGqiA8vkIkVAFgIELiFeLkFeLk=iY=Hhbbf9v8qqaqFr0xc9pk0xbba9q8WqFfeaY=biLkVcLq=JHqVepeea0=as0db9vqpepesP0xe9Fve9Fve9GapdbaqaaeGacaGaaiaabeqaamqadiabaaGcbaGafmOCaiNbaSaaaaa@3E6A@_0 _= (*x*_0_, *y*_0_, *z*_0_), representing the heart's electrical activity, in a conductive half space, representing the torso. The coordinate system is chosen such that z = 0 at the boundary between the "torso" with constant conductivity and the non-conducting space containing the measurement sites.

The magnetic flux density at coordinate r→
 MathType@MTEF@5@5@+=feaafiart1ev1aaatCvAUfKttLearuWrP9MDH5MBPbIqV92AaeXatLxBI9gBaebbnrfifHhDYfgasaacH8akY=wiFfYdH8Gipec8Eeeu0xXdbba9frFj0=OqFfea0dXdd9vqai=hGuQ8kuc9pgc9s8qqaq=dirpe0xb9q8qiLsFr0=vr0=vr0dc8meaabaqaciaacaGaaeqabaqabeGadaaakeaacuWGYbGCgaWcaaaa@2E2B@ = (*x*, *y*, *z*) above the half space (z > 0) is according to [32] given by

B→(r→)=μ04π K2{[(p→×R→)e→z]∇K−K e→z×p→}     (5)
 MathType@MTEF@5@5@+=feaafiart1ev1aaatCvAUfKttLearuWrP9MDH5MBPbIqV92AaeXatLxBI9gBaebbnrfifHhDYfgasaacH8akY=wiFfYdH8Gipec8Eeeu0xXdbba9frFj0=OqFfea0dXdd9vqai=hGuQ8kuc9pgc9s8qqaq=dirpe0xb9q8qiLsFr0=vr0=vr0dc8meaabaqaciaacaGaaeqabaqabeGadaaakeaacuWGcbGqgaWcaiabcIcaOiqbdkhaYzaalaGaeiykaKIaeyypa0ZaaSaaaeaaiiGacqWF8oqBdaWgaaWcbaGaeGimaadabeaaaOqaaiabisda0iab=b8aWjabbccaGiabdUealnaaCaaaleqabaGaeGOmaidaaaaakmaacmqabaWaamWaaeaadaqadaqaaiqbdchaWzaalaGaey41aqRafmOuaiLbaSaaaiaawIcacaGLPaaacuWGLbqzgaWcamaaBaaaleaacqWG6bGEaeqaaaGccaGLBbGaayzxaaGaey4bIeTaem4saSKaeyOeI0Iaem4saSKaeeiiaaIafmyzauMbaSaadaWgaaWcbaGaemOEaOhabeaakiabgEna0kqbdchaWzaalaaacaGL7bGaayzFaaGaaCzcaiaaxMaadaqadaqaaiabiwda1aGaayjkaiaawMcaaaaa@582D@

with R→
 MathType@MTEF@5@5@+=feaafiart1ev1aaatCvAUfKttLearuWrP9MDH5MBPbIqV92AaeXatLxBI9gBaebbnrfifHhDYfgasaacH8akY=wiFfYdH8Gipec8Eeeu0xXdbba9frFj0=OqFfea0dXdd9vqai=hGuQ8kuc9pgc9s8qqaq=dirpe0xb9q8qiLsFr0=vr0=vr0dc8meaabaqaciaacaGaaeqabaqabeGadaaakeaacuWGsbGugaWcaaaa@2DEB@ = r→
 MathType@MTEF@5@5@+=feaafiart1ev1aaatCvAUfKttLearuWrP9MDH5MBPbIqV92AaeXatLxBI9gBaebbnrfifHhDYfgasaacH8akY=wiFfYdH8Gipec8Eeeu0xXdbba9frFj0=OqFfea0dXdd9vqai=hGuQ8kuc9pgc9s8qqaq=dirpe0xb9q8qiLsFr0=vr0=vr0dc8meaabaqaciaacaGaaeqabaqabeGadaaakeaacuWGYbGCgaWcaaaa@2E2B@ - r→
 MathType@MTEF@5@5@+=feaafiart1ev1aaatCvAUfKttLearuWrP9MDH5MBPbIqV92AaeXatLxBI9gBaebbnrfifHhDYfgasaacH8akY=wiFfYdH8Gipec8Eeeu0xXdbba9frFj0=OqFfea0dXdd9vqai=hGuQ8kuc9pgc9s8qqaq=dirpe0xb9q8qiLsFr0=vr0=vr0dc8meaabaqaciaacaGaaeqabaqabeGadaaakeaacuWGYbGCgaWcaaaa@2E2B@_0_, *R *= |R→
 MathType@MTEF@5@5@+=feaafiart1ev1aaatCvAUfKttLearuWrP9MDH5MBPbIqV92AaeXatLxBI9gBaebbnrfifHhDYfgasaacH8akY=wiFfYdH8Gipec8Eeeu0xXdbba9frFj0=OqFfea0dXdd9vqai=hGuQ8kuc9pgc9s8qqaq=dirpe0xb9q8qiLsFr0=vr0=vr0dc8meaabaqaciaacaGaaeqabaqabeGadaaakeaacuWGsbGugaWcaaaa@2DEB@|, *K *= *R *(*R *+ R→
 MathType@MTEF@5@5@+=feaafiart1ev1aaatCvAUfKttLearuWrP9MDH5MBPbIqV92AaeXatLxBI9gBaebbnrfifHhDYfgasaacH8akY=wiFfYdH8Gipec8Eeeu0xXdbba9frFj0=OqFfea0dXdd9vqai=hGuQ8kuc9pgc9s8qqaq=dirpe0xb9q8qiLsFr0=vr0=vr0dc8meaabaqaciaacaGaaeqabaqabeGadaaakeaacuWGsbGugaWcaaaa@2DEB@e→
 MathType@MTEF@5@5@+=feaafiart1ev1aaatCvAUfKttLearuWrP9MDH5MBPbIqV92AaeXatLxBI9gBaebbnrfifHhDYfgasaacH8akY=wiFfYdH8Gipec8Eeeu0xXdbba9frFj0=OqFfea0dXdd9vqai=hGuQ8kuc9pgc9s8qqaq=dirpe0xb9q8qiLsFr0=vr0=vr0dc8meaabaqaciaacaGaaeqabaqabeGadaaakeaacuWGLbqzgaWcaaaa@2E11@_*z*_), and ∇ K=(2+R→ e→zR)R→+Re→z
 MathType@MTEF@5@5@+=feaafiart1ev1aaatCvAUfKttLearuWrP9MDH5MBPbIqV92AaeXatLxBI9gBaebbnrfifHhDYfgasaacH8akY=wiFfYdH8Gipec8Eeeu0xXdbba9frFj0=OqFfea0dXdd9vqai=hGuQ8kuc9pgc9s8qqaq=dirpe0xb9q8qiLsFr0=vr0=vr0dc8meaabaqaciaacaGaaeqabaqabeGadaaakeaacqGHhis0cqqGGaaicqWGlbWscqGH9aqpcqGGOaakcqaIYaGmcqGHRaWkdaWcaaqaaiqbdkfaszaalaGaeeiiaaIafmyzauMbaSaadaWgaaWcbaGaemOEaOhabeaaaOqaaiabdkfasbaacqGGPaqkcuWGsbGugaWcaiabgUcaRiabdkfasjqbcwgaLzaalaWaaSbaaSqaaiabdQha6bqabaaaaa@415A@, where ∇ is the nabla operator. In Cartesian coordinates B→
 MathType@MTEF@5@5@+=feaafiart1ev1aaatCvAUfKttLearuWrP9MDH5MBPbIqV92AaeXatLxBI9gBamXvP5wqSXMqHnxAJn0BKvguHDwzZbqegyvzYrwyUfgarqqtubsr4rNCHbGeaGqiA8vkIkVAFgIELiFeLkFeLk=iY=Hhbbf9v8qqaqFr0xc9pk0xbba9q8WqFfeaY=biLkVcLq=JHqVepeea0=as0db9vqpepesP0xe9Fve9Fve9GapdbaqaaeGacaGaaiaabeqaamqadiabaaGcbaGafmOqaiKbaSaaaaa@3E0A@ can be explicitly written as

Bx(r→)=μ04πpxXYX2+Y2[2X2+Y2(1−ZR)−ZR3]+μ04πpy1X2+Y2[Y2−X2X2+Y2(1−ZR)+X2ZR3],     (6)
 MathType@MTEF@5@5@+=feaafiart1ev1aaatCvAUfKttLearuWrP9MDH5MBPbIqV92AaeXatLxBI9gBamXvP5wqSXMqHnxAJn0BKvguHDwzZbqegyvzYrwyUfgarqqtubsr4rNCHbGeaGqiA8vkIkVAFgIELiFeLkFeLk=iY=Hhbbf9v8qqaqFr0xc9pk0xbba9q8WqFfeaY=biLkVcLq=JHqVepeea0=as0db9vqpepesP0xe9Fve9Fve9GapdbaqaaeGacaGaaiaabeqaamqadiabaaGcbaqbaeWabiqaaaqaaiabdkeacnaaBaaaleaacqWG4baEaeqaaOWaaeWaaeaacuWGYbGCgaWcaaGaayjkaiaawMcaaiabg2da9maalaaabaacciGae8hVd02aaSbaaSqaaiabicdaWaqabaaakeaacqaI0aancqWFapaCaaGaemiCaa3aaSbaaSqaaiabdIha4bqabaGcdaWcaaqaaiabdIfayjabdMfazbqaaiabdIfaynaaCaaaleqabaGaeGOmaidaaOGaey4kaSIaemywaK1aaWbaaSqabeaacqaIYaGmaaaaaOWaamWaaeaadaWcaaqaaiabikdaYaqaaiabdIfaynaaCaaaleqabaGaeGOmaidaaOGaey4kaSIaemywaK1aaWbaaSqabeaacqaIYaGmaaaaaOWaaeWaaeaacqaIXaqmcqGHsisldaWcaaqaaiabdQfaAbqaaiabdkfasbaaaiaawIcacaGLPaaacqGHsisldaWcaaqaaiabdQfaAbqaaiabdkfasnaaCaaaleqabaGaeG4mamdaaaaaaOGaay5waiaaw2faaaqaaiabgUcaRmaalaaabaGae8hVd02aaSbaaSqaaiabicdaWaqabaaakeaacqaI0aancqWFapaCaaGaemiCaa3aaSbaaSqaaiabdMha5bqabaGcdaWcaaqaaiabigdaXaqaaiabdIfaynaaCaaaleqabaGaeGOmaidaaOGaey4kaSIaemywaK1aaWbaaSqabeaacqaIYaGmaaaaaOWaamWaaeaadaWcaaqaaiabdMfaznaaCaaaleqabaGaeGOmaidaaOGaeyOeI0IaemiwaG1aaWbaaSqabeaacqaIYaGmaaaakeaacqWGybawdaahaaWcbeqaaiabikdaYaaakiabgUcaRiabdMfaznaaCaaaleqabaGaeGOmaidaaaaakmaabmaabaGaeGymaeJaeyOeI0YaaSaaaeaacqWGAbGwaeaacqWGsbGuaaaacaGLOaGaayzkaaGaey4kaSYaaSaaaeaacqWGybawdaahaaWcbeqaaiabikdaYaaakiabdQfaAbqaaiabdkfasnaaCaaaleqabaGaeG4mamdaaaaaaOGaay5waiaaw2faaiabcYcaSaaacaWLjaGaaCzcamaabmaabaGaeGOnaydacaGLOaGaayzkaaaaaa@96B9@

By(r→)=μ04πpx1X2+Y2[Y2−X2X2+Y2(1−ZR)−Y2ZR3]             −μ04πpyXYX2+Y2[2X2+Y2(1−ZR)−ZR3],     (7)
 MathType@MTEF@5@5@+=feaafiart1ev1aaatCvAUfKttLearuWrP9MDH5MBPbIqV92AaeXatLxBI9gBamXvP5wqSXMqHnxAJn0BKvguHDwzZbqegyvzYrwyUfgarqqtubsr4rNCHbGeaGqiA8vkIkVAFgIELiFeLkFeLk=iY=Hhbbf9v8qqaqFr0xc9pk0xbba9q8WqFfeaY=biLkVcLq=JHqVepeea0=as0db9vqpepesP0xe9Fve9Fve9GapdbaqaaeGacaGaaiaabeqaamqadiabaaGcbaqbaeWabiqaaaqaaiabdkeacnaaBaaaleaacqWG5bqEaeqaaOWaaeWaaeaacuWGYbGCgaWcaaGaayjkaiaawMcaaiabg2da9maalaaabaacciGae8hVd02aaSbaaSqaaiabicdaWaqabaaakeaacqaI0aancqWFapaCaaGaemiCaa3aaSbaaSqaaiabdIha4bqabaGcdaWcaaqaaiabigdaXaqaaiabdIfaynaaCaaaleqabaGaeGOmaidaaOGaey4kaSIaemywaK1aaWbaaSqabeaacqaIYaGmaaaaaOWaamWaaeaadaWcaaqaaiabdMfaznaaCaaaleqabaGaeGOmaidaaOGaeyOeI0IaemiwaG1aaWbaaSqabeaacqaIYaGmaaaakeaacqWGybawdaahaaWcbeqaaiabikdaYaaakiabgUcaRiabdMfaznaaCaaaleqabaGaeGOmaidaaaaakmaabmaabaGaeGymaeJaeyOeI0YaaSaaaeaacqWGAbGwaeaacqWGsbGuaaaacaGLOaGaayzkaaGaeyOeI0YaaSaaaeaacqWGzbqwdaahaaWcbeqaaiabikdaYaaakiabdQfaAbqaaiabdkfasnaaCaaaleqabaGaeG4mamdaaaaaaOGaay5waiaaw2faaaqaaiaaywW6caaMfSUaaGzbRlabgkHiTmaalaaabaGae8hVd02aaSbaaSqaaiabicdaWaqabaaakeaacqaI0aancqWFapaCaaGaemiCaa3aaSbaaSqaaiabdMha5bqabaGcdaWcaaqaaiabdIfayjabdMfazbqaaiabdIfaynaaCaaaleqabaGaeGOmaidaaOGaey4kaSIaemywaK1aaWbaaSqabeaacqaIYaGmaaaaaOWaamWaaeaadaWcaaqaaiabikdaYaqaaiabdIfaynaaCaaaleqabaGaeGOmaidaaOGaey4kaSIaemywaK1aaWbaaSqabeaacqaIYaGmaaaaaOWaaeWaaeaacqaIXaqmcqGHsisldaWcaaqaaiabdQfaAbqaaiabdkfasbaaaiaawIcacaGLPaaacqGHsisldaWcaaqaaiabdQfaAbqaaiabdkfasnaaCaaaleqabaGaeG4mamdaaaaaaOGaay5waiaaw2faaiabcYcaSaaacaWLjaGaaCzcamaabmaabaGaeG4naCdacaGLOaGaayzkaaaaaa@9B73@

Bz(r→)=μ04πYpx−XpyR3     (8)
 MathType@MTEF@5@5@+=feaafiart1ev1aaatCvAUfKttLearuWrP9MDH5MBPbIqV92AaeXatLxBI9gBaebbnrfifHhDYfgasaacH8akY=wiFfYdH8Gipec8Eeeu0xXdbba9frFj0=OqFfea0dXdd9vqai=hGuQ8kuc9pgc9s8qqaq=dirpe0xb9q8qiLsFr0=vr0=vr0dc8meaabaqaciaacaGaaeqabaqabeGadaaakeaacqWGcbGqdaWgaaWcbaGaemOEaOhabeaakmaabmaabaGafmOCaiNbaSaaaiaawIcacaGLPaaacqGH9aqpdaWcaaqaaGGaciab=X7aTnaaBaaaleaacqaIWaamaeqaaaGcbaGaeGinaqJae8hWdahaamaalaaabaGaemywaKLaemiCaa3aaSbaaSqaaiabdIha4bqabaGccqGHsislcqWGybawcqWGWbaCdaWgaaWcbaGaemyEaKhabeaaaOqaaiabdkfasnaaCaaaleqabaGaeG4mamdaaaaakiaaxMaacaWLjaWaaeWaaeaacqaI4aaoaiaawIcacaGLPaaaaaa@48DF@

with *X *= (*x *- *x*_0_), *Y *= (*y *- *y*_0_) and *Z *= (*z *- *z*_0_).

An inspection of (6)–(8) shows that

• *p*_*z *_does not contribute to B→
 MathType@MTEF@5@5@+=feaafiart1ev1aaatCvAUfKttLearuWrP9MDH5MBPbIqV92AaeXatLxBI9gBamXvP5wqSXMqHnxAJn0BKvguHDwzZbqegyvzYrwyUfgarqqtubsr4rNCHbGeaGqiA8vkIkVAFgIELiFeLkFeLk=iY=Hhbbf9v8qqaqFr0xc9pk0xbba9q8WqFfeaY=biLkVcLq=JHqVepeea0=as0db9vqpepesP0xe9Fve9Fve9GapdbaqaaeGacaGaaiaabeqaamqadiabaaGcbaGafmOqaiKbaSaaaaa@3E0A@(r→
 MathType@MTEF@5@5@+=feaafiart1ev1aaatCvAUfKttLearuWrP9MDH5MBPbIqV92AaeXatLxBI9gBamXvP5wqSXMqHnxAJn0BKvguHDwzZbqegyvzYrwyUfgarqqtubsr4rNCHbGeaGqiA8vkIkVAFgIELiFeLkFeLk=iY=Hhbbf9v8qqaqFr0xc9pk0xbba9q8WqFfeaY=biLkVcLq=JHqVepeea0=as0db9vqpepesP0xe9Fve9Fve9GapdbaqaaeGacaGaaiaabeqaamqadiabaaGcbaGafmOCaiNbaSaaaaa@3E6A@) above z > 0,

• *B*_*x*_(r→
 MathType@MTEF@5@5@+=feaafiart1ev1aaatCvAUfKttLearuWrP9MDH5MBPbIqV92AaeXatLxBI9gBamXvP5wqSXMqHnxAJn0BKvguHDwzZbqegyvzYrwyUfgarqqtubsr4rNCHbGeaGqiA8vkIkVAFgIELiFeLkFeLk=iY=Hhbbf9v8qqaqFr0xc9pk0xbba9q8WqFfeaY=biLkVcLq=JHqVepeea0=as0db9vqpepesP0xe9Fve9Fve9GapdbaqaaeGacaGaaiaabeqaamqadiabaaGcbaGafmOCaiNbaSaaaaa@3E6A@), *B*_*y*_(r→
 MathType@MTEF@5@5@+=feaafiart1ev1aaatCvAUfKttLearuWrP9MDH5MBPbIqV92AaeXatLxBI9gBamXvP5wqSXMqHnxAJn0BKvguHDwzZbqegyvzYrwyUfgarqqtubsr4rNCHbGeaGqiA8vkIkVAFgIELiFeLkFeLk=iY=Hhbbf9v8qqaqFr0xc9pk0xbba9q8WqFfeaY=biLkVcLq=JHqVepeea0=as0db9vqpepesP0xe9Fve9Fve9GapdbaqaaeGacaGaaiaabeqaamqadiabaaGcbaGafmOCaiNbaSaaaaa@3E6A@), and *B*_*z*_(r→
 MathType@MTEF@5@5@+=feaafiart1ev1aaatCvAUfKttLearuWrP9MDH5MBPbIqV92AaeXatLxBI9gBamXvP5wqSXMqHnxAJn0BKvguHDwzZbqegyvzYrwyUfgarqqtubsr4rNCHbGeaGqiA8vkIkVAFgIELiFeLkFeLk=iY=Hhbbf9v8qqaqFr0xc9pk0xbba9q8WqFfeaY=biLkVcLq=JHqVepeea0=as0db9vqpepesP0xe9Fve9Fve9GapdbaqaaeGacaGaaiaabeqaamqadiabaaGcbaGafmOCaiNbaSaaaaa@3E6A@) do not depend on the position of the torso boundary as long as it is between measurement point and current dipole,

• the difference to the B→
 MathType@MTEF@5@5@+=feaafiart1ev1aaatCvAUfKttLearuWrP9MDH5MBPbIqV92AaeXatLxBI9gBamXvP5wqSXMqHnxAJn0BKvguHDwzZbqegyvzYrwyUfgarqqtubsr4rNCHbGeaGqiA8vkIkVAFgIELiFeLkFeLk=iY=Hhbbf9v8qqaqFr0xc9pk0xbba9q8WqFfeaY=biLkVcLq=JHqVepeea0=as0db9vqpepesP0xe9Fve9Fve9GapdbaqaaeGacaGaaiaabeqaamqadiabaaGcbaGafmOqaiKbaSaaaaa@3E0A@ – field calculated by Biot-Savart's law for an isolated current dipole occurs only in (6) and (7),

• B→
 MathType@MTEF@5@5@+=feaafiart1ev1aaatCvAUfKttLearuWrP9MDH5MBPbIqV92AaeXatLxBI9gBamXvP5wqSXMqHnxAJn0BKvguHDwzZbqegyvzYrwyUfgarqqtubsr4rNCHbGeaGqiA8vkIkVAFgIELiFeLkFeLk=iY=Hhbbf9v8qqaqFr0xc9pk0xbba9q8WqFfeaY=biLkVcLq=JHqVepeea0=as0db9vqpepesP0xe9Fve9Fve9GapdbaqaaeGacaGaaiaabeqaamqadiabaaGcbaGafmOqaiKbaSaaaaa@3E0A@(r→
 MathType@MTEF@5@5@+=feaafiart1ev1aaatCvAUfKttLearuWrP9MDH5MBPbIqV92AaeXatLxBI9gBamXvP5wqSXMqHnxAJn0BKvguHDwzZbqegyvzYrwyUfgarqqtubsr4rNCHbGeaGqiA8vkIkVAFgIELiFeLkFeLk=iY=Hhbbf9v8qqaqFr0xc9pk0xbba9q8WqFfeaY=biLkVcLq=JHqVepeea0=as0db9vqpepesP0xe9Fve9Fve9GapdbaqaaeGacaGaaiaabeqaamqadiabaaGcbaGafmOCaiNbaSaaaaa@3E6A@) is independent of the value of the constant conductivity in the half space.

Note that the above field properties are also valid for a horizontally layered conductor, i.e. for a conductivity σ = σ (*z*).

Now the Hosaka-Cohen transformation (3) is applied to (8) and yields

c→=μ04π[pxe→x+pye→yR3−3{[pxY2−pyXY]e→x+[pyX2−pxXY]e→y}R5].     (9)
 MathType@MTEF@5@5@+=feaafiart1ev1aaatCvAUfKttLearuWrP9MDH5MBPbIqV92AaeXatLxBI9gBaebbnrfifHhDYfgasaacH8akY=wiFfYdH8Gipec8Eeeu0xXdbba9frFj0=OqFfea0dXdd9vqai=hGuQ8kuc9pgc9s8qqaq=dirpe0xb9q8qiLsFr0=vr0=vr0dc8meaabaqaciaacaGaaeqabaqabeGadaaakeaacuWGJbWygaWcaiabg2da9maalaaabaacciGae8hVd02aaSbaaSqaaiabicdaWaqabaaakeaacqaI0aancqWFapaCaaWaamWaaeaadaWcaaqaaiabdchaWnaaBaaaleaacqWG4baEaeqaaOGafmyzauMbaSaadaWgaaWcbaGaemiEaGhabeaakiabgUcaRiabdchaWnaaBaaaleaacqWG5bqEaeqaaOGafmyzauMbaSaadaWgaaWcbaGaemyEaKhabeaaaOqaaiabdkfasnaaCaaaleqabaGaeG4mamdaaaaakiabgkHiTmaalaaabaGaeG4mamZaaiWabeaadaWadaqaaiabdchaWnaaBaaaleaacqWG4baEaeqaaOGaemywaK1aaWbaaSqabeaacqaIYaGmaaGccqGHsislcqWGWbaCdaWgaaWcbaGaemyEaKhabeaakiabdIfayjabdMfazbGaay5waiaaw2faaiqbdwgaLzaalaWaaSbaaSqaaiabdIha4bqabaGccqGHRaWkdaWadaqaaiabdchaWnaaBaaaleaacqWG5bqEaeqaaOGaemiwaG1aaWbaaSqabeaacqaIYaGmaaGccqGHsislcqWGWbaCdaWgaaWcbaGaemiEaGhabeaakiabdIfayjabdMfazbGaay5waiaaw2faaiqbdwgaLzaalaWaaSbaaSqaaiabdMha5bqabaaakiaawUhacaGL9baaaeaacqWGsbGudaahaaWcbeqaaiabiwda1aaaaaaakiaawUfacaGLDbaacqGGUaGlcaWLjaGaaCzcamaabmaabaGaeGyoaKdacaGLOaGaayzkaaaaaa@7456@

Particularly for *X *= 0, *Y *= 0, i.e. directly above the current dipole, one obtains

c→=μ04πpxe→x+pye→yZ3.     (10)
 MathType@MTEF@5@5@+=feaafiart1ev1aaatCvAUfKttLearuWrP9MDH5MBPbIqV92AaeXatLxBI9gBaebbnrfifHhDYfgasaacH8akY=wiFfYdH8Gipec8Eeeu0xXdbba9frFj0=OqFfea0dXdd9vqai=hGuQ8kuc9pgc9s8qqaq=dirpe0xb9q8qiLsFr0=vr0=vr0dc8meaabaqaciaacaGaaeqabaqabeGadaaakeaacuWGJbWygaWcaiabg2da9maalaaabaacciGae8hVd02aaSbaaSqaaiabicdaWaqabaaakeaacqaI0aancqWFapaCaaWaaSaaaeaacqWGWbaCdaWgaaWcbaGaemiEaGhabeaakiqbdwgaLzaalaWaaSbaaSqaaiabdIha4bqabaGccqGHRaWkcqWGWbaCdaWgaaWcbaGaemyEaKhabeaakiqbdwgaLzaalaWaaSbaaSqaaiabdMha5bqabaaakeaacqWGAbGwdaahaaWcbeqaaiabiodaZaaaaaGccqGGUaGlcaWLjaGaaCzcamaabmaabaGaeGymaeJaeGimaadacaGLOaGaayzkaaaaaa@49F7@

In this case c→
 MathType@MTEF@5@5@+=feaafiart1ev1aaatCvAUfKttLearuWrP9MDH5MBPbIqV92AaeXatLxBI9gBaebbnrfifHhDYfgasaacH8akY=wiFfYdH8Gipec8Eeeu0xXdbba9frFj0=OqFfea0dXdd9vqai=hGuQ8kuc9pgc9s8qqaq=dirpe0xb9q8qiLsFr0=vr0=vr0dc8meaabaqaciaacaGaaeqabaqabeGadaaakeaacuWGJbWygaWcaaaa@2E0D@ is directly proportional to the *x*-*y*-projection of the current dipole moment p→
 MathType@MTEF@5@5@+=feaafiart1ev1aaatCvAUfKttLearuWrP9MDH5MBPbIqV92AaeXatLxBI9gBaebbnrfifHhDYfgasaacH8akY=wiFfYdH8Gipec8Eeeu0xXdbba9frFj0=OqFfea0dXdd9vqai=hGuQ8kuc9pgc9s8qqaq=dirpe0xb9q8qiLsFr0=vr0=vr0dc8meaabaqaciaacaGaaeqabaqabeGadaaakeaacuWGWbaCgaWcaaaa@2E27@.

This supports the argument that the Hosaka-Cohen transformation is really related to the underlying current source. However, it is also evident from (9) that additional terms are blurring and distorting the image.

On first sight the distribution of arrows might suggest that this is an image not only of the current dipole but also of the return currents (also termed: volume currents). And indeed, the model "dipole current in a conductive half space" considers the role of the return currents. However, in this special geometry, the volume currents do not contribute to *B*_*z*_(r→
 MathType@MTEF@5@5@+=feaafiart1ev1aaatCvAUfKttLearuWrP9MDH5MBPbIqV92AaeXatLxBI9gBaebbnrfifHhDYfgasaacH8akY=wiFfYdH8Gipec8Eeeu0xXdbba9frFj0=OqFfea0dXdd9vqai=hGuQ8kuc9pgc9s8qqaq=dirpe0xb9q8qiLsFr0=vr0=vr0dc8meaabaqaciaacaGaaeqabaqabeGadaaakeaacuWGYbGCgaWcaaaa@2E2B@)as can be seen above. It becomes also evident, that the spatial distribution of c→
 MathType@MTEF@5@5@+=feaafiart1ev1aaatCvAUfKttLearuWrP9MDH5MBPbIqV92AaeXatLxBI9gBaebbnrfifHhDYfgasaacH8akY=wiFfYdH8Gipec8Eeeu0xXdbba9frFj0=OqFfea0dXdd9vqai=hGuQ8kuc9pgc9s8qqaq=dirpe0xb9q8qiLsFr0=vr0=vr0dc8meaabaqaciaacaGaaeqabaqabeGadaaakeaacuWGJbWygaWcaaaa@2E0D@ away from *X *= 0, *Y *= 0 does not represent the return currents if *Z *is varied. Without loss in validity of equations (6)–(8) one may consider that p→
 MathType@MTEF@5@5@+=feaafiart1ev1aaatCvAUfKttLearuWrP9MDH5MBPbIqV92AaeXatLxBI9gBaebbnrfifHhDYfgasaacH8akY=wiFfYdH8Gipec8Eeeu0xXdbba9frFj0=OqFfea0dXdd9vqai=hGuQ8kuc9pgc9s8qqaq=dirpe0xb9q8qiLsFr0=vr0=vr0dc8meaabaqaciaacaGaaeqabaqabeGadaaakeaacuWGWbaCgaWcaaaa@2E27@ is very close to the half space interface *z *= 0 and the measurement of *B*_*z*_(*x*, *y*) is performed at different distances approaching p→
 MathType@MTEF@5@5@+=feaafiart1ev1aaatCvAUfKttLearuWrP9MDH5MBPbIqV92AaeXatLxBI9gBaebbnrfifHhDYfgasaacH8akY=wiFfYdH8Gipec8Eeeu0xXdbba9frFj0=OqFfea0dXdd9vqai=hGuQ8kuc9pgc9s8qqaq=dirpe0xb9q8qiLsFr0=vr0=vr0dc8meaabaqaciaacaGaaeqabaqabeGadaaakeaacuWGWbaCgaWcaaaa@2E27@. In this theoretical case, the image approximates in the limit (*z *- *z*_0_) = 0 a point-like distribution with vanishing c→
 MathType@MTEF@5@5@+=feaafiart1ev1aaatCvAUfKttLearuWrP9MDH5MBPbIqV92AaeXatLxBI9gBaebbnrfifHhDYfgasaacH8akY=wiFfYdH8Gipec8Eeeu0xXdbba9frFj0=OqFfea0dXdd9vqai=hGuQ8kuc9pgc9s8qqaq=dirpe0xb9q8qiLsFr0=vr0=vr0dc8meaabaqaciaacaGaaeqabaqabeGadaaakeaacuWGJbWygaWcaaaa@2E0D@(*x*, *y*) apart from the origin *X *= 0, *Y *= 0. However, the volume currents keep their amplitude independently from *z *as only the measurement device is moved and not the current dipole source. Thus the nature of the c→
 MathType@MTEF@5@5@+=feaafiart1ev1aaatCvAUfKttLearuWrP9MDH5MBPbIqV92AaeXatLxBI9gBaebbnrfifHhDYfgasaacH8akY=wiFfYdH8Gipec8Eeeu0xXdbba9frFj0=OqFfea0dXdd9vqai=hGuQ8kuc9pgc9s8qqaq=dirpe0xb9q8qiLsFr0=vr0=vr0dc8meaabaqaciaacaGaaeqabaqabeGadaaakeaacuWGJbWygaWcaaaa@2E0D@ – image is a point spread function of non-radial symmetry.

A closer look to just one component (e.g. the *x*-component) of c→
 MathType@MTEF@5@5@+=feaafiart1ev1aaatCvAUfKttLearuWrP9MDH5MBPbIqV92AaeXatLxBI9gBaebbnrfifHhDYfgasaacH8akY=wiFfYdH8Gipec8Eeeu0xXdbba9frFj0=OqFfea0dXdd9vqai=hGuQ8kuc9pgc9s8qqaq=dirpe0xb9q8qiLsFr0=vr0=vr0dc8meaabaqaciaacaGaaeqabaqabeGadaaakeaacuWGJbWygaWcaaaa@2E0D@ reveals that it is composed of two terms

cx=∂Bz∂y=μ04π[pxR3−3Y[Ypx−Xpy]R5].             (11)
 MathType@MTEF@5@5@+=feaafiart1ev1aaatCvAUfKttLearuWrP9MDH5MBPbIqV92AaeXatLxBI9gBaebbnrfifHhDYfgasaacH8akY=wiFfYdH8Gipec8Eeeu0xXdbba9frFj0=OqFfea0dXdd9vqai=hGuQ8kuc9pgc9s8qqaq=dirpe0xb9q8qiLsFr0=vr0=vr0dc8meaabaqaciaacaGaaeqabaqabeGadaaakeaacqWGJbWydaWgaaWcbaGaemiEaGhabeaakiabg2da9maalaaabaGaeyOaIyRaemOqai0aaSbaaSqaaiabdQha6bqabaaakeaacqGHciITcqWG5bqEaaGaeyypa0ZaaSaaaeaacqaH8oqBdaWgaaWcbaGaeGimaadabeaaaOqaaiabisda0iabec8aWbaadaWadaqaamaalaaabaGaemiCaa3aaSbaaSqaaiabdIha4bqabaaakeaacqWGsbGudaahaaWcbeqaaiabiodaZaaaaaGccqGHsisldaWcaaqaaiabiodaZiabdMfaznaadmaabaGaemywaKLaemiCaa3aaSbaaSqaaiabdIha4bqabaGccqGHsislcqWGybawcqWGWbaCdaWgaaWcbaGaemyEaKhabeaaaOGaay5waiaaw2faaaqaaiabdkfasnaaCaaaleqabaGaeGynaudaaaaaaOGaay5waiaaw2faaiabc6caUiabbccaGiabbccaGiabbccaGiabbccaGiabbccaGiabbccaGiabbccaGiabbccaGiabbccaGiabbccaGiabbccaGiabbccaGiabbccaGiabbIcaOiabbgdaXiabbgdaXiabbMcaPaaa@656A@

**Figure 6:**
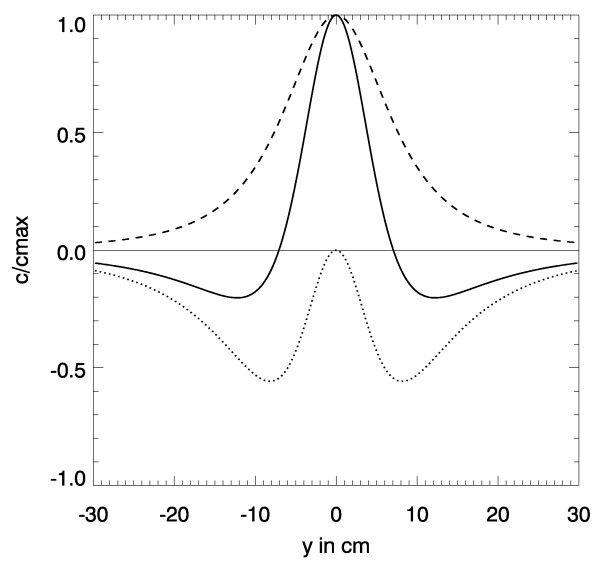
Spatial dependence of |c→
 MathType@MTEF@5@5@+=feaafiart1ev1aaatCvAUfKttLearuWrP9MDH5MBPbIqV92AaeXatLxBI9gBaebbnrfifHhDYfgasaacH8akY=wiFfYdH8Gipec8Eeeu0xXdbba9frFj0=OqFfea0dXdd9vqai=hGuQ8kuc9pgc9s8qqaq=dirpe0xb9q8qiLsFr0=vr0=vr0dc8meaabaqaciaacaGaaeqabaqabeGadaaakeaacuWGJbWygaWcaaaa@2E0D@_*p*_| along the symmetry axis orthogonal to the dipole direction. Dashed line: refers to the first term in eq. (11); dotted line: refers to the second term in eq. (11); solid line: both terms. Same data as in Fig. 5, however the direction of the dipole is in *x*-direction.

The spatial distribution of both terms is shown in Fig. 6 as a solid line. While the first term – shown as a dotted line – is radially symmetric the second term is not. Along the symmetry axis parallel to the direction of the dipole this latter term is vanishing, see the dotted line in Fig. 6. Unfortunately the second term is of the same order of magnitude as the first term. Thus *c*_*x *_is not directly proportional to *p*_*x *_as the second term contains mixed terms. However, it contributes a kind of focussing effect.

#### Current dipole in a conductive sphere

For a current dipole p→
 MathType@MTEF@5@5@+=feaafiart1ev1aaatCvAUfKttLearuWrP9MDH5MBPbIqV92AaeXatLxBI9gBaebbnrfifHhDYfgasaacH8akY=wiFfYdH8Gipec8Eeeu0xXdbba9frFj0=OqFfea0dXdd9vqai=hGuQ8kuc9pgc9s8qqaq=dirpe0xb9q8qiLsFr0=vr0=vr0dc8meaabaqaciaacaGaaeqabaqabeGadaaakeaacuWGWbaCgaWcaaaa@2E27@ at r→
 MathType@MTEF@5@5@+=feaafiart1ev1aaatCvAUfKttLearuWrP9MDH5MBPbIqV92AaeXatLxBI9gBaebbnrfifHhDYfgasaacH8akY=wiFfYdH8Gipec8Eeeu0xXdbba9frFj0=OqFfea0dXdd9vqai=hGuQ8kuc9pgc9s8qqaq=dirpe0xb9q8qiLsFr0=vr0=vr0dc8meaabaqaciaacaGaaeqabaqabeGadaaakeaacuWGYbGCgaWcaaaa@2E2B@_0 _in a conductive sphere similar relations can be obtained in terms of spherical coordinates *r*, ϑ, φ. The magnetic flux density outside the sphere is given by [32]

B→(r→)=μ04πF2{F(p→×r→0)−[(p→×r→0)⋅r→]∇F}     (12)
 MathType@MTEF@5@5@+=feaafiart1ev1aaatCvAUfKttLearuWrP9MDH5MBPbIqV92AaeXatLxBI9gBaebbnrfifHhDYfgasaacH8akY=wiFfYdH8Gipec8Eeeu0xXdbba9frFj0=OqFfea0dXdd9vqai=hGuQ8kuc9pgc9s8qqaq=dirpe0xb9q8qiLsFr0=vr0=vr0dc8meaabaqaciaacaGaaeqabaqabeGadaaakeaacuWGcbGqgaWcaiabcIcaOiqbdkhaYzaalaGaeiykaKIaeyypa0ZaaSaaaeaaiiGacqWF8oqBdaWgaaWcbaGaeGimaadabeaaaOqaaiabisda0iab=b8aWjabdAeagnaaCaaaleqabaGaeGOmaidaaaaakmaacmqabaGaemOrayKaeiikaGIafmiCaaNbaSaacqGHxdaTcuWGYbGCgaWcamaaBaaaleaacqaIWaamaeqaaOGaeiykaKIaeyOeI0YaamWaaeaacqGGOaakcuWGWbaCgaWcaiabgEna0kqbdkhaYzaalaWaaSbaaSqaaiabicdaWaqabaGccqGGPaqkcqGHflY1cuWGYbGCgaWcaaGaay5waiaaw2faaiabgEGirlabdAeagbGaay5Eaiaaw2haaiaaxMaacaWLjaWaaeWaaeaacqaIXaqmcqaIYaGmaiaawIcacaGLPaaaaaa@5AE6@

with *F *= *R*(*r R *+ r→
 MathType@MTEF@5@5@+=feaafiart1ev1aaatCvAUfKttLearuWrP9MDH5MBPbIqV92AaeXatLxBI9gBaebbnrfifHhDYfgasaacH8akY=wiFfYdH8Gipec8Eeeu0xXdbba9frFj0=OqFfea0dXdd9vqai=hGuQ8kuc9pgc9s8qqaq=dirpe0xb9q8qiLsFr0=vr0=vr0dc8meaabaqaciaacaGaaeqabaqabeGadaaakeaacuWGYbGCgaWcaaaa@2E2B@R→
 MathType@MTEF@5@5@+=feaafiart1ev1aaatCvAUfKttLearuWrP9MDH5MBPbIqV92AaeXatLxBI9gBaebbnrfifHhDYfgasaacH8akY=wiFfYdH8Gipec8Eeeu0xXdbba9frFj0=OqFfea0dXdd9vqai=hGuQ8kuc9pgc9s8qqaq=dirpe0xb9q8qiLsFr0=vr0=vr0dc8meaabaqaciaacaGaaeqabaqabeGadaaakeaacuWGsbGugaWcaaaa@2DEB@), R→
 MathType@MTEF@5@5@+=feaafiart1ev1aaatCvAUfKttLearuWrP9MDH5MBPbIqV92AaeXatLxBI9gBaebbnrfifHhDYfgasaacH8akY=wiFfYdH8Gipec8Eeeu0xXdbba9frFj0=OqFfea0dXdd9vqai=hGuQ8kuc9pgc9s8qqaq=dirpe0xb9q8qiLsFr0=vr0=vr0dc8meaabaqaciaacaGaaeqabaqabeGadaaakeaacuWGsbGugaWcaaaa@2DEB@ = r→
 MathType@MTEF@5@5@+=feaafiart1ev1aaatCvAUfKttLearuWrP9MDH5MBPbIqV92AaeXatLxBI9gBaebbnrfifHhDYfgasaacH8akY=wiFfYdH8Gipec8Eeeu0xXdbba9frFj0=OqFfea0dXdd9vqai=hGuQ8kuc9pgc9s8qqaq=dirpe0xb9q8qiLsFr0=vr0=vr0dc8meaabaqaciaacaGaaeqabaqabeGadaaakeaacuWGYbGCgaWcaaaa@2E2B@ - r→
 MathType@MTEF@5@5@+=feaafiart1ev1aaatCvAUfKttLearuWrP9MDH5MBPbIqV92AaeXatLxBI9gBaebbnrfifHhDYfgasaacH8akY=wiFfYdH8Gipec8Eeeu0xXdbba9frFj0=OqFfea0dXdd9vqai=hGuQ8kuc9pgc9s8qqaq=dirpe0xb9q8qiLsFr0=vr0=vr0dc8meaabaqaciaacaGaaeqabaqabeGadaaakeaacuWGYbGCgaWcaaaa@2E2B@_0_, *R *= |R→
 MathType@MTEF@5@5@+=feaafiart1ev1aaatCvAUfKttLearuWrP9MDH5MBPbIqV92AaeXatLxBI9gBaebbnrfifHhDYfgasaacH8akY=wiFfYdH8Gipec8Eeeu0xXdbba9frFj0=OqFfea0dXdd9vqai=hGuQ8kuc9pgc9s8qqaq=dirpe0xb9q8qiLsFr0=vr0=vr0dc8meaabaqaciaacaGaaeqabaqabeGadaaakeaacuWGsbGugaWcaaaa@2DEB@|, *r *= |r→
 MathType@MTEF@5@5@+=feaafiart1ev1aaatCvAUfKttLearuWrP9MDH5MBPbIqV92AaeXatLxBI9gBaebbnrfifHhDYfgasaacH8akY=wiFfYdH8Gipec8Eeeu0xXdbba9frFj0=OqFfea0dXdd9vqai=hGuQ8kuc9pgc9s8qqaq=dirpe0xb9q8qiLsFr0=vr0=vr0dc8meaabaqaciaacaGaaeqabaqabeGadaaakeaacuWGYbGCgaWcaaaa@2E2B@|, and

∇*F *= [*r*^-1 ^*R*^2 ^+ *R*^-1 ^(r→
 MathType@MTEF@5@5@+=feaafiart1ev1aaatCvAUfKttLearuWrP9MDH5MBPbIqV92AaeXatLxBI9gBaebbnrfifHhDYfgasaacH8akY=wiFfYdH8Gipec8Eeeu0xXdbba9frFj0=OqFfea0dXdd9vqai=hGuQ8kuc9pgc9s8qqaq=dirpe0xb9q8qiLsFr0=vr0=vr0dc8meaabaqaciaacaGaaeqabaqabeGadaaakeaacuWGYbGCgaWcaaaa@2E2B@R→
 MathType@MTEF@5@5@+=feaafiart1ev1aaatCvAUfKttLearuWrP9MDH5MBPbIqV92AaeXatLxBI9gBaebbnrfifHhDYfgasaacH8akY=wiFfYdH8Gipec8Eeeu0xXdbba9frFj0=OqFfea0dXdd9vqai=hGuQ8kuc9pgc9s8qqaq=dirpe0xb9q8qiLsFr0=vr0=vr0dc8meaabaqaciaacaGaaeqabaqabeGadaaakeaacuWGsbGugaWcaaaa@2DEB@) + 2*R *+2*r*]r→
 MathType@MTEF@5@5@+=feaafiart1ev1aaatCvAUfKttLearuWrP9MDH5MBPbIqV92AaeXatLxBI9gBaebbnrfifHhDYfgasaacH8akY=wiFfYdH8Gipec8Eeeu0xXdbba9frFj0=OqFfea0dXdd9vqai=hGuQ8kuc9pgc9s8qqaq=dirpe0xb9q8qiLsFr0=vr0=vr0dc8meaabaqaciaacaGaaeqabaqabeGadaaakeaacuWGYbGCgaWcaaaa@2E2B@ - [*R *+ 2*r *+ *R*^-1 ^(r→
 MathType@MTEF@5@5@+=feaafiart1ev1aaatCvAUfKttLearuWrP9MDH5MBPbIqV92AaeXatLxBI9gBaebbnrfifHhDYfgasaacH8akY=wiFfYdH8Gipec8Eeeu0xXdbba9frFj0=OqFfea0dXdd9vqai=hGuQ8kuc9pgc9s8qqaq=dirpe0xb9q8qiLsFr0=vr0=vr0dc8meaabaqaciaacaGaaeqabaqabeGadaaakeaacuWGYbGCgaWcaaaa@2E2B@R→
 MathType@MTEF@5@5@+=feaafiart1ev1aaatCvAUfKttLearuWrP9MDH5MBPbIqV92AaeXatLxBI9gBaebbnrfifHhDYfgasaacH8akY=wiFfYdH8Gipec8Eeeu0xXdbba9frFj0=OqFfea0dXdd9vqai=hGuQ8kuc9pgc9s8qqaq=dirpe0xb9q8qiLsFr0=vr0=vr0dc8meaabaqaciaacaGaaeqabaqabeGadaaakeaacuWGsbGugaWcaaaa@2DEB@)]r→
 MathType@MTEF@5@5@+=feaafiart1ev1aaatCvAUfKttLearuWrP9MDH5MBPbIqV92AaeXatLxBI9gBaebbnrfifHhDYfgasaacH8akY=wiFfYdH8Gipec8Eeeu0xXdbba9frFj0=OqFfea0dXdd9vqai=hGuQ8kuc9pgc9s8qqaq=dirpe0xb9q8qiLsFr0=vr0=vr0dc8meaabaqaciaacaGaaeqabaqabeGadaaakeaacuWGYbGCgaWcaaaa@2E2B@_0_.

This expression is valid for a conductivity profile σ = σ (*r*).

For the case of the dipole being positioned on the *z*-axis at r→
 MathType@MTEF@5@5@+=feaafiart1ev1aaatCvAUfKttLearuWrP9MDH5MBPbIqV92AaeXatLxBI9gBaebbnrfifHhDYfgasaacH8akY=wiFfYdH8Gipec8Eeeu0xXdbba9frFj0=OqFfea0dXdd9vqai=hGuQ8kuc9pgc9s8qqaq=dirpe0xb9q8qiLsFr0=vr0=vr0dc8meaabaqaciaacaGaaeqabaqabeGadaaakeaacuWGYbGCgaWcaaaa@2E2B@_0 _= (0, 0, *z*_0_) the radial component of B→
 MathType@MTEF@5@5@+=feaafiart1ev1aaatCvAUfKttLearuWrP9MDH5MBPbIqV92AaeXatLxBI9gBamXvP5wqSXMqHnxAJn0BKvguHDwzZbqegyvzYrwyUfgarqqtubsr4rNCHbGeaGqiA8vkIkVAFgIELiFeLkFeLk=iY=Hhbbf9v8qqaqFr0xc9pk0xbba9q8WqFfeaY=biLkVcLq=JHqVepeea0=as0db9vqpepesP0xe9Fve9Fve9GapdbaqaaeGacaGaaiaabeqaamqadiabaaGcbaGafmOqaiKbaSaaaaa@3E0A@(r→
 MathType@MTEF@5@5@+=feaafiart1ev1aaatCvAUfKttLearuWrP9MDH5MBPbIqV92AaeXatLxBI9gBamXvP5wqSXMqHnxAJn0BKvguHDwzZbqegyvzYrwyUfgarqqtubsr4rNCHbGeaGqiA8vkIkVAFgIELiFeLkFeLk=iY=Hhbbf9v8qqaqFr0xc9pk0xbba9q8WqFfeaY=biLkVcLq=JHqVepeea0=as0db9vqpepesP0xe9Fve9Fve9GapdbaqaaeGacaGaaiaabeqaamqadiabaaGcbaGafmOCaiNbaSaaaaa@3E6A@) becomes

Br=μ04πz0sin⁡ϑ (pxsin⁡ϕ−pycos⁡ϕ)R3     (13)
 MathType@MTEF@5@5@+=feaafiart1ev1aaatCvAUfKttLearuWrP9MDH5MBPbIqV92AaeXatLxBI9gBamXvP5wqSXMqHnxAJn0BKvguHDwzZbqegyvzYrwyUfgarqqtubsr4rNCHbGeaGqiA8vkIkVAFgIELiFeLkFeLk=iY=Hhbbf9v8qqaqFr0xc9pk0xbba9q8WqFfeaY=biLkVcLq=JHqVepeea0=as0db9vqpepesP0xe9Fve9Fve9GapdbaqaaeGacaGaaiaabeqaamqadiabaaGcbaGaemOqai0aaSbaaSqaaiabdkhaYbqabaGccqGH9aqpdaWcaaqaaGGaciab=X7aTnaaBaaaleaacqaIWaamaeqaaaGcbaGaeGinaqJae8hWdahaamaalaaabaGaemOEaO3aaSbaaSqaaiabicdaWaqabaGccyGGZbWCcqGGPbqAcqGGUbGBcqaHrpGscqqGGaaicqGGOaakcqWGWbaCdaWgaaWcbaGaemiEaGhabeaakiGbcohaZjabcMgaPjabc6gaUjab=v9aQjabgkHiTiabdchaWnaaBaaaleaacqWG5bqEaeqaaOGagi4yamMaei4Ba8Maei4CamxcaaMae8x1dOMccqGGPaqkaeaacqWGsbGudaahaaWcbeqaaiabiodaZaaaaaGccaWLjaGaaCzcamaabmaabaGaeGymaeJaeG4mamdacaGLOaGaayzkaaaaaa@6BBB@

with *R *= (*r*^2 ^- 2 *z*_0 _*r *cosϑ + z02
 MathType@MTEF@5@5@+=feaafiart1ev1aaatCvAUfKttLearuWrP9MDH5MBPbIqV92AaeXatLxBI9gBaebbnrfifHhDYfgasaacH8akY=wiFfYdH8Gipec8Eeeu0xXdbba9frFj0=OqFfea0dXdd9vqai=hGuQ8kuc9pgc9s8qqaq=dirpe0xb9q8qiLsFr0=vr0=vr0dc8meaabaqaciaacaGaaeqabaqabeGadaaakeaacqWG6bGEdaqhaaWcbaGaeGimaadabaGaeGOmaidaaaaa@3036@)^1/2^.

Then for the pseudo current density the following relation

c→=1rsin⁡ϑ∂Br∂ϕe→ϑ−1r∂Br∂ϑe→ϕ     (14)
 MathType@MTEF@5@5@+=feaafiart1ev1aaatCvAUfKttLearuWrP9MDH5MBPbIqV92AaeXatLxBI9gBaebbnrfifHhDYfgasaacH8akY=wiFfYdH8Gipec8Eeeu0xXdbba9frFj0=OqFfea0dXdd9vqai=hGuQ8kuc9pgc9s8qqaq=dirpe0xb9q8qiLsFr0=vr0=vr0dc8meaabaqaciaacaGaaeqabaqabeGadaaakeaacuWGJbWygaWcaiabg2da9maalaaabaGaeGymaedabaGaemOCaiNagi4CamNaeiyAaKMaeiOBa4Maeqy0dOeaamaalaaabaGaeyOaIyRaemOqai0aaSbaaSqaaiabdkhaYbqabaaakeaacqGHciITiiGacqWFvpGAaaGafmyzauMbaSaadaWgaaWcbaGaeqy0dOeabeaakiabgkHiTmaalaaabaGaeGymaedabaGaemOCaihaamaalaaabaGaeyOaIyRaemOqai0aaSbaaSqaaiabdkhaYbqabaaakeaacqGHciITcqaHrpGsaaGafmyzauMbaSaadaWgaaWcbaGae8x1dOgabeaakiaaxMaacaWLjaWaaeWaaeaacqaIXaqmcqaI0aanaiaawIcacaGLPaaaaaa@549B@

gained from curl B→
 MathType@MTEF@5@5@+=feaafiart1ev1aaatCvAUfKttLearuWrP9MDH5MBPbIqV92AaeXatLxBI9gBamXvP5wqSXMqHnxAJn0BKvguHDwzZbqegyvzYrwyUfgarqqtubsr4rNCHbGeaGqiA8vkIkVAFgIELiFeLkFeLk=iY=Hhbbf9v8qqaqFr0xc9pk0xbba9q8WqFfeaY=biLkVcLq=JHqVepeea0=as0db9vqpepesP0xe9Fve9Fve9GapdbaqaaeGacaGaaiaabeqaamqadiabaaGcbaGafmOqaiKbaSaaaaa@3E0A@ in spherical coordinates may be applied to (13) leading to

c→=μ04πz0r R3[(pxcos⁡ϕ+pysin⁡ϕ)e→ϑ−(pxsin⁡ϕ−pycos⁡ϕ)(cos⁡ϑ−3z0rsin⁡2ϑR2)e→ϕ]     (15)
 MathType@MTEF@5@5@+=feaafiart1ev1aaatCvAUfKttLearuWrP9MDH5MBPbIqV92AaeXatLxBI9gBaebbnrfifHhDYfgasaacH8akY=wiFfYdH8Gipec8Eeeu0xXdbba9frFj0=OqFfea0dXdd9vqai=hGuQ8kuc9pgc9s8qqaq=dirpe0xb9q8qiLsFr0=vr0=vr0dc8meaabaqaciaacaGaaeqabaqabeGadaaakeaacuWGJbWygaWcaiabg2da9maalaaabaacciGae8hVd02aaSbaaSqaaiabicdaWaqabaaakeaacqaI0aancqWFapaCaaWaaSaaaeaacqWG6bGEdaWgaaWcbaGaeGimaadabeaaaOqaaiabdkhaYjabbccaGiabdkfasnaaCaaaleqabaGaeG4mamdaaaaakmaadmaabaWaaeWaaeaacqWGWbaCdaWgaaWcbaGaemiEaGhabeaakiGbcogaJjabc+gaVjabcohaZjab=v9aQjabgUcaRiabdchaWnaaBaaaleaacqWG5bqEaeqaaOGagi4CamNaeiyAaKMaeiOBa4Mae8x1dOgacaGLOaGaayzkaaGafmyzauMbaSaadaWgaaWcbaGaeqy0dOeabeaakiabgkHiTmaabmaabaGaemiCaa3aaSbaaSqaaiabdIha4bqabaGccyGGZbWCcqGGPbqAcqGGUbGBcqWFvpGAcqGHsislcqWGWbaCdaWgaaWcbaGaemyEaKhabeaakiGbcogaJjabc+gaVjabcohaZjab=v9aQbGaayjkaiaawMcaamaabmaabaGagi4yamMaei4Ba8Maei4CamNaeqy0dOKaeyOeI0YaaSaaaeaacqaIZaWmcqWG6bGEdaWgaaWcbaGaeGimaadabeaakiabdkhaYjGbcohaZjabcMgaPjabc6gaUnaaCaaaleqabaGaeGOmaidaaOGaeqy0dOeabaGaemOuai1aaWbaaSqabeaacqaIYaGmaaaaaaGccaGLOaGaayzkaaGafmyzauMbaSaadaWgaaWcbaGae8x1dOgabeaaaOGaay5waiaaw2faaiaaxMaacaWLjaWaaeWaaeaacqaIXaqmcqaI1aqnaiaawIcacaGLPaaaaaa@89D6@

Particularly for ϑ = 0, i. e. directly above the current dipole, one obtains

c→=μ04πz0z(z−z0)3(pxe→x+pye→y).     (16)
 MathType@MTEF@5@5@+=feaafiart1ev1aaatCvAUfKttLearuWrP9MDH5MBPbIqV92AaeXatLxBI9gBaebbnrfifHhDYfgasaacH8akY=wiFfYdH8Gipec8Eeeu0xXdbba9frFj0=OqFfea0dXdd9vqai=hGuQ8kuc9pgc9s8qqaq=dirpe0xb9q8qiLsFr0=vr0=vr0dc8meaabaqaciaacaGaaeqabaqabeGadaaakeaacuWGJbWygaWcaiabg2da9maalaaabaacciGae8hVd02aaSbaaSqaaiabicdaWaqabaaakeaacqaI0aancqWFapaCaaWaaSaaaeaacqWG6bGEdaWgaaWcbaGaeGimaadabeaaaOqaaiabdQha6jabcIcaOiabdQha6jabgkHiTiabdQha6naaBaaaleaacqaIWaamaeqaaOGaeiykaKYaaWbaaSqabeaacqaIZaWmaaaaaOWaaeWaaeaacqWGWbaCdaWgaaWcbaGaemiEaGhabeaakiqbdwgaLzaalaWaaSbaaSqaaiabdIha4bqabaGccqGHRaWkcqWGWbaCdaWgaaWcbaGaemyEaKhabeaakiqbdwgaLzaalaWaaSbaaSqaaiabdMha5bqabaaakiaawIcacaGLPaaacqGGUaGlcaWLjaGaaCzcamaabmaabaGaeGymaeJaeGOnaydacaGLOaGaayzkaaaaaa@552A@

Thus the discussion of the results follows the same lines as in the preceding chapter.

### Pseudo current density maps for MNG and MEG recordings

**Figure 7:**
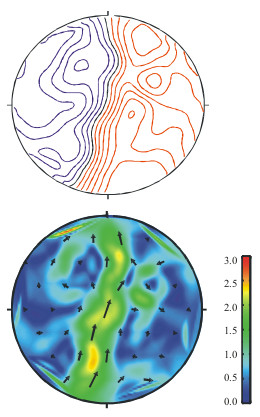
Top: *B*_*z*_-map of an electrically stimulated nerve signal recorded over the lumbar spine (contour step 1fT), and bottom: its HC-transformation (scale in fT/cm).

In Fig. 7 isocontour and PCD-maps of an MNG recording using 49 channels of a planar SQUID system are shown. The centre of the system was placed over the lumbar spine with a distance of approximately 8 cm between the magnetic sensors and leg nerves coming from the left leg entering the spine. The nerve response to electrical stimulation at the ankle with amplitude of about 10 mA and duration of 100 μs was recorded. 9.000 responses were averaged to improve the pure signal-to-noise ratio. In Fig. 7, top, an isocontour map of the *B*_*z*_-field component 15 ms after the stimulus and in Fig. 7, bottom, the corresponding PCD-map are shown. Inspecting the isocontour map from Fig. 7 only a raw understanding of an underlying current and its direction corresponding to the zero line of the map is possible for an expert. The PCD-map allows a more intuitive conclusion that the underlying nerve current is extremely extended and slightly curved.

**Figure 8:**
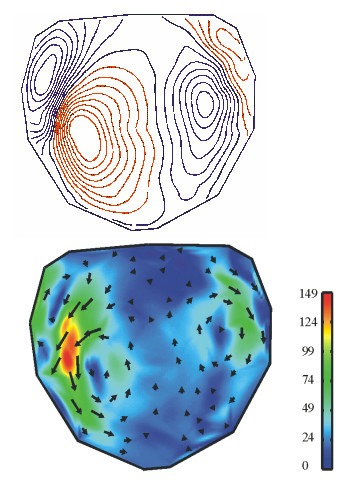
Top: *B*_*r*_-map of an acoustically stimulated (1 kHz) N100 signal (contour step 50fT), and bottom: its HC-transformation (scale in fT/cm).

Fig. 8 displays maps of an acoustically evoked MEG recorded in a helmet system with 93 channels. The spherical maps are unfolded, the nose is situated at the top, and ears are at the right and left side, respectively. The measurement recorded the brain response to acoustic stimulation with a 1 kHz sinusoidal tone. 30 stimuli were averaged. In Fig. 8, top, an isocontour map of the radial field component at the occurrence of the maximum of the response (about 100 ms after stimulus; termed "N100") is shown and in Fig. 8, bottom, the corresponding PCD-map. Using the isocontour map from Fig. 8 the number of sources and their configuration cannot be concluded. On the other hand from inspecting the PCD-map one can conclude that two separate focal sources are active, one in each hemisphere in the corresponding acoustic cortex.

## Discussion

### Alternative pseudo current density maps and corresponding hardware realizations

**Figure 9:**
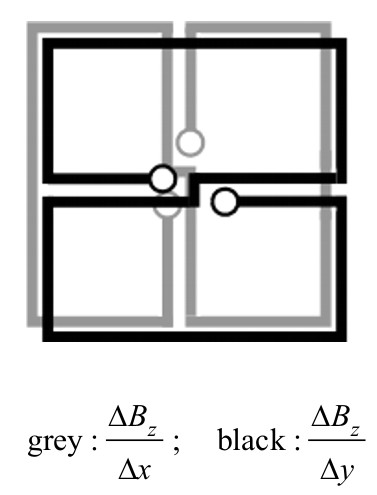
Planar *B*_*z*_-gradiometer as a hardware realization for performing direct HC-transformations and the related difference quotients.

The Hosaka-Cohen transformation is nothing else but a combination of partial derivatives of components of B→
 MathType@MTEF@5@5@+=feaafiart1ev1aaatCvAUfKttLearuWrP9MDH5MBPbIqV92AaeXatLxBI9gBamXvP5wqSXMqHnxAJn0BKvguHDwzZbqegyvzYrwyUfgarqqtubsr4rNCHbGeaGqiA8vkIkVAFgIELiFeLkFeLk=iY=Hhbbf9v8qqaqFr0xc9pk0xbba9q8WqFfeaY=biLkVcLq=JHqVepeea0=as0db9vqpepesP0xe9Fve9Fve9GapdbaqaaeGacaGaaiaabeqaamqadiabaaGcbaGafmOqaiKbaSaaaaa@3E0A@(r→
 MathType@MTEF@5@5@+=feaafiart1ev1aaatCvAUfKttLearuWrP9MDH5MBPbIqV92AaeXatLxBI9gBamXvP5wqSXMqHnxAJn0BKvguHDwzZbqegyvzYrwyUfgarqqtubsr4rNCHbGeaGqiA8vkIkVAFgIELiFeLkFeLk=iY=Hhbbf9v8qqaqFr0xc9pk0xbba9q8WqFfeaY=biLkVcLq=JHqVepeea0=as0db9vqpepesP0xe9Fve9Fve9GapdbaqaaeGacaGaaiaabeqaamqadiabaaGcbaGafmOCaiNbaSaaaaa@3E6A@). Planar gradiometers are hardware realizations that provide an approximation of the partial derivative of B→
 MathType@MTEF@5@5@+=feaafiart1ev1aaatCvAUfKttLearuWrP9MDH5MBPbIqV92AaeXatLxBI9gBamXvP5wqSXMqHnxAJn0BKvguHDwzZbqegyvzYrwyUfgarqqtubsr4rNCHbGeaGqiA8vkIkVAFgIELiFeLkFeLk=iY=Hhbbf9v8qqaqFr0xc9pk0xbba9q8WqFfeaY=biLkVcLq=JHqVepeea0=as0db9vqpepesP0xe9Fve9Fve9GapdbaqaaeGacaGaaiaabeqaamqadiabaaGcbaGafmOqaiKbaSaaaaa@3E0A@(r→
 MathType@MTEF@5@5@+=feaafiart1ev1aaatCvAUfKttLearuWrP9MDH5MBPbIqV92AaeXatLxBI9gBamXvP5wqSXMqHnxAJn0BKvguHDwzZbqegyvzYrwyUfgarqqtubsr4rNCHbGeaGqiA8vkIkVAFgIELiFeLkFeLk=iY=Hhbbf9v8qqaqFr0xc9pk0xbba9q8WqFfeaY=biLkVcLq=JHqVepeea0=as0db9vqpepesP0xe9Fve9Fve9GapdbaqaaeGacaGaaiaabeqaamqadiabaaGcbaGafmOCaiNbaSaaaaa@3E6A@). Thus, the SQUID-chip introduced by [33], which is a combination of *x*- and *y*-gradiometers, provides -if wired accordingly- just the approximation of c→
 MathType@MTEF@5@5@+=feaafiart1ev1aaatCvAUfKttLearuWrP9MDH5MBPbIqV92AaeXatLxBI9gBaebbnrfifHhDYfgasaacH8akY=wiFfYdH8Gipec8Eeeu0xXdbba9frFj0=OqFfea0dXdd9vqai=hGuQ8kuc9pgc9s8qqaq=dirpe0xb9q8qiLsFr0=vr0=vr0dc8meaabaqaciaacaGaaeqabaqabeGadaaakeaacuWGJbWygaWcaaaa@2E0D@(*x*, *y*) (cf. Fig. 9). Consequently, the software of the first SQUID-systems of that design contained a program called "arrow mapper".

As mentioned before an interrelation between c→
 MathType@MTEF@5@5@+=feaafiart1ev1aaatCvAUfKttLearuWrP9MDH5MBPbIqV92AaeXatLxBI9gBaebbnrfifHhDYfgasaacH8akY=wiFfYdH8Gipec8Eeeu0xXdbba9frFj0=OqFfea0dXdd9vqai=hGuQ8kuc9pgc9s8qqaq=dirpe0xb9q8qiLsFr0=vr0=vr0dc8meaabaqaciaacaGaaeqabaqabeGadaaakeaacuWGJbWygaWcaaaa@2E0D@ and curl B→
 MathType@MTEF@5@5@+=feaafiart1ev1aaatCvAUfKttLearuWrP9MDH5MBPbIqV92AaeXatLxBI9gBamXvP5wqSXMqHnxAJn0BKvguHDwzZbqegyvzYrwyUfgarqqtubsr4rNCHbGeaGqiA8vkIkVAFgIELiFeLkFeLk=iY=Hhbbf9v8qqaqFr0xc9pk0xbba9q8WqFfeaY=biLkVcLq=JHqVepeea0=as0db9vqpepesP0xe9Fve9Fve9GapdbaqaaeGacaGaaiaabeqaamqadiabaaGcbaGafmOqaiKbaSaaaaa@3E0A@ exists. Equation (4) may be rewritten as

curl B→=(∂Bx∂ze→y−∂Bx∂ye→z)+(∂By∂xe→z−∂By∂ze→x)+(∂Bz∂ye→x−∂Bz∂xe→y).     (17)
 MathType@MTEF@5@5@+=feaafiart1ev1aaatCvAUfKttLearuWrP9MDH5MBPbIqV92AaeXatLxBI9gBaebbnrfifHhDYfgasaacH8akY=wiFfYdH8Gipec8Eeeu0xXdbba9frFj0=OqFfea0dXdd9vqai=hGuQ8kuc9pgc9s8qqaq=dirpe0xb9q8qiLsFr0=vr0=vr0dc8meaabaqaciaacaGaaeqabaqabeGadaaakeaacqqGJbWycqqG1bqDcqqGYbGCcqqGSbaBcqqGGaaicuWGcbGqgaWcaiabg2da9maabmaabaWaaSaaaeaacqGHciITcqWGcbGqdaWgaaWcbaGaemiEaGhabeaaaOqaaiabgkGi2kabdQha6baacuWGLbqzgaWcamaaBaaaleaacqWG5bqEaeqaaOGaeyOeI0YaaSaaaeaacqGHciITcqWGcbGqdaWgaaWcbaGaemiEaGhabeaaaOqaaiabgkGi2kabdMha5baacuWGLbqzgaWcamaaBaaaleaacqWG6bGEaeqaaaGccaGLOaGaayzkaaGaey4kaSYaaeWaaeaadaWcaaqaaiabgkGi2kabdkeacnaaBaaaleaacqWG5bqEaeqaaaGcbaGaeyOaIyRaemiEaGhaaiqbdwgaLzaalaWaaSbaaSqaaiabdQha6bqabaGccqGHsisldaWcaaqaaiabgkGi2kabdkeacnaaBaaaleaacqWG5bqEaeqaaaGcbaGaeyOaIyRaemOEaOhaaiqbdwgaLzaalaWaaSbaaSqaaiabdIha4bqabaaakiaawIcacaGLPaaacqGHRaWkdaqadaqaamaalaaabaGaeyOaIyRaemOqai0aaSbaaSqaaiabdQha6bqabaaakeaacqGHciITcqWG5bqEaaGafmyzauMbaSaadaWgaaWcbaGaemiEaGhabeaakiabgkHiTmaalaaabaGaeyOaIyRaemOqai0aaSbaaSqaaiabdQha6bqabaaakeaacqGHciITcqWG4baEaaGafmyzauMbaSaadaWgaaWcbaGaemyEaKhabeaaaOGaayjkaiaawMcaaiabc6caUiaaxMaacaWLjaWaaeWaaeaacqaIXaqmcqaI3aWnaiaawIcacaGLPaaaaaa@80E5@

For this case curl B→
 MathType@MTEF@5@5@+=feaafiart1ev1aaatCvAUfKttLearuWrP9MDH5MBPbIqV92AaeXatLxBI9gBamXvP5wqSXMqHnxAJn0BKvguHDwzZbqegyvzYrwyUfgarqqtubsr4rNCHbGeaGqiA8vkIkVAFgIELiFeLkFeLk=iY=Hhbbf9v8qqaqFr0xc9pk0xbba9q8WqFfeaY=biLkVcLq=JHqVepeea0=as0db9vqpepesP0xe9Fve9Fve9GapdbaqaaeGacaGaaiaabeqaamqadiabaaGcbaGafmOqaiKbaSaaaaa@3E0A@ can be written as a sum of three vectors a→
 MathType@MTEF@5@5@+=feaafiart1ev1aaatCvAUfKttLearuWrP9MDH5MBPbIqV92AaeXatLxBI9gBamXvP5wqSXMqHnxAJn0BKvguHDwzZbqegyvzYrwyUfgarqqtubsr4rNCHbGeaGqiA8vkIkVAFgIELiFeLkFeLk=iY=Hhbbf9v8qqaqFr0xc9pk0xbba9q8WqFfeaY=biLkVcLq=JHqVepeea0=as0db9vqpepesP0xe9Fve9Fve9GapdbaqaaeGacaGaaiaabeqaamqadiabaaGcbaGafmyyaeMbaSaaaaa@3E48@, b→
 MathType@MTEF@5@5@+=feaafiart1ev1aaatCvAUfKttLearuWrP9MDH5MBPbIqV92AaeXatLxBI9gBamXvP5wqSXMqHnxAJn0BKvguHDwzZbqegyvzYrwyUfgarqqtubsr4rNCHbGeaGqiA8vkIkVAFgIELiFeLkFeLk=iY=Hhbbf9v8qqaqFr0xc9pk0xbba9q8WqFfeaY=biLkVcLq=JHqVepeea0=as0db9vqpepesP0xe9Fve9Fve9GapdbaqaaeGacaGaaiaabeqaamqadiabaaGcbaGafmOyaiMbaSaaaaa@3E4A@, and c→
 MathType@MTEF@5@5@+=feaafiart1ev1aaatCvAUfKttLearuWrP9MDH5MBPbIqV92AaeXatLxBI9gBaebbnrfifHhDYfgasaacH8akY=wiFfYdH8Gipec8Eeeu0xXdbba9frFj0=OqFfea0dXdd9vqai=hGuQ8kuc9pgc9s8qqaq=dirpe0xb9q8qiLsFr0=vr0=vr0dc8meaabaqaciaacaGaaeqabaqabeGadaaakeaacuWGJbWygaWcaaaa@2E0D@ where c→
 MathType@MTEF@5@5@+=feaafiart1ev1aaatCvAUfKttLearuWrP9MDH5MBPbIqV92AaeXatLxBI9gBaebbnrfifHhDYfgasaacH8akY=wiFfYdH8Gipec8Eeeu0xXdbba9frFj0=OqFfea0dXdd9vqai=hGuQ8kuc9pgc9s8qqaq=dirpe0xb9q8qiLsFr0=vr0=vr0dc8meaabaqaciaacaGaaeqabaqabeGadaaakeaacuWGJbWygaWcaaaa@2E0D@ is identical to (3), i.e.

curl B→
 MathType@MTEF@5@5@+=feaafiart1ev1aaatCvAUfKttLearuWrP9MDH5MBPbIqV92AaeXatLxBI9gBamXvP5wqSXMqHnxAJn0BKvguHDwzZbqegyvzYrwyUfgarqqtubsr4rNCHbGeaGqiA8vkIkVAFgIELiFeLkFeLk=iY=Hhbbf9v8qqaqFr0xc9pk0xbba9q8WqFfeaY=biLkVcLq=JHqVepeea0=as0db9vqpepesP0xe9Fve9Fve9GapdbaqaaeGacaGaaiaabeqaamqadiabaaGcbaGafmOqaiKbaSaaaaa@3E0A@ = a→
 MathType@MTEF@5@5@+=feaafiart1ev1aaatCvAUfKttLearuWrP9MDH5MBPbIqV92AaeXatLxBI9gBamXvP5wqSXMqHnxAJn0BKvguHDwzZbqegyvzYrwyUfgarqqtubsr4rNCHbGeaGqiA8vkIkVAFgIELiFeLkFeLk=iY=Hhbbf9v8qqaqFr0xc9pk0xbba9q8WqFfeaY=biLkVcLq=JHqVepeea0=as0db9vqpepesP0xe9Fve9Fve9GapdbaqaaeGacaGaaiaabeqaamqadiabaaGcbaGafmyyaeMbaSaaaaa@3E48@ + b→
 MathType@MTEF@5@5@+=feaafiart1ev1aaatCvAUfKttLearuWrP9MDH5MBPbIqV92AaeXatLxBI9gBamXvP5wqSXMqHnxAJn0BKvguHDwzZbqegyvzYrwyUfgarqqtubsr4rNCHbGeaGqiA8vkIkVAFgIELiFeLkFeLk=iY=Hhbbf9v8qqaqFr0xc9pk0xbba9q8WqFfeaY=biLkVcLq=JHqVepeea0=as0db9vqpepesP0xe9Fve9Fve9GapdbaqaaeGacaGaaiaabeqaamqadiabaaGcbaGafmOyaiMbaSaaaaa@3E4A@ + c→
 MathType@MTEF@5@5@+=feaafiart1ev1aaatCvAUfKttLearuWrP9MDH5MBPbIqV92AaeXatLxBI9gBaebbnrfifHhDYfgasaacH8akY=wiFfYdH8Gipec8Eeeu0xXdbba9frFj0=OqFfea0dXdd9vqai=hGuQ8kuc9pgc9s8qqaq=dirpe0xb9q8qiLsFr0=vr0=vr0dc8meaabaqaciaacaGaaeqabaqabeGadaaakeaacuWGJbWygaWcaaaa@2E0D@     (18)

with .

Outside the body a→
 MathType@MTEF@5@5@+=feaafiart1ev1aaatCvAUfKttLearuWrP9MDH5MBPbIqV92AaeXatLxBI9gBamXvP5wqSXMqHnxAJn0BKvguHDwzZbqegyvzYrwyUfgarqqtubsr4rNCHbGeaGqiA8vkIkVAFgIELiFeLkFeLk=iY=Hhbbf9v8qqaqFr0xc9pk0xbba9q8WqFfeaY=biLkVcLq=JHqVepeea0=as0db9vqpepesP0xe9Fve9Fve9GapdbaqaaeGacaGaaiaabeqaamqadiabaaGcbaGafmyyaeMbaSaaaaa@3E48@ + b→
 MathType@MTEF@5@5@+=feaafiart1ev1aaatCvAUfKttLearuWrP9MDH5MBPbIqV92AaeXatLxBI9gBamXvP5wqSXMqHnxAJn0BKvguHDwzZbqegyvzYrwyUfgarqqtubsr4rNCHbGeaGqiA8vkIkVAFgIELiFeLkFeLk=iY=Hhbbf9v8qqaqFr0xc9pk0xbba9q8WqFfeaY=biLkVcLq=JHqVepeea0=as0db9vqpepesP0xe9Fve9Fve9GapdbaqaaeGacaGaaiaabeqaamqadiabaaGcbaGafmOyaiMbaSaaaaa@3E4A@ + c→
 MathType@MTEF@5@5@+=feaafiart1ev1aaatCvAUfKttLearuWrP9MDH5MBPbIqV92AaeXatLxBI9gBaebbnrfifHhDYfgasaacH8akY=wiFfYdH8Gipec8Eeeu0xXdbba9frFj0=OqFfea0dXdd9vqai=hGuQ8kuc9pgc9s8qqaq=dirpe0xb9q8qiLsFr0=vr0=vr0dc8meaabaqaciaacaGaaeqabaqabeGadaaakeaacuWGJbWygaWcaaaa@2E0D@ = 0 due to curl B→
 MathType@MTEF@5@5@+=feaafiart1ev1aaatCvAUfKttLearuWrP9MDH5MBPbIqV92AaeXatLxBI9gBamXvP5wqSXMqHnxAJn0BKvguHDwzZbqegyvzYrwyUfgarqqtubsr4rNCHbGeaGqiA8vkIkVAFgIELiFeLkFeLk=iY=Hhbbf9v8qqaqFr0xc9pk0xbba9q8WqFfeaY=biLkVcLq=JHqVepeea0=as0db9vqpepesP0xe9Fve9Fve9GapdbaqaaeGacaGaaiaabeqaamqadiabaaGcbaGafmOqaiKbaSaaaaa@3E0A@ = 0. Therefore a→
 MathType@MTEF@5@5@+=feaafiart1ev1aaatCvAUfKttLearuWrP9MDH5MBPbIqV92AaeXatLxBI9gBamXvP5wqSXMqHnxAJn0BKvguHDwzZbqegyvzYrwyUfgarqqtubsr4rNCHbGeaGqiA8vkIkVAFgIELiFeLkFeLk=iY=Hhbbf9v8qqaqFr0xc9pk0xbba9q8WqFfeaY=biLkVcLq=JHqVepeea0=as0db9vqpepesP0xe9Fve9Fve9GapdbaqaaeGacaGaaiaabeqaamqadiabaaGcbaGafmyyaeMbaSaaaaa@3E48@ + b→
 MathType@MTEF@5@5@+=feaafiart1ev1aaatCvAUfKttLearuWrP9MDH5MBPbIqV92AaeXatLxBI9gBamXvP5wqSXMqHnxAJn0BKvguHDwzZbqegyvzYrwyUfgarqqtubsr4rNCHbGeaGqiA8vkIkVAFgIELiFeLkFeLk=iY=Hhbbf9v8qqaqFr0xc9pk0xbba9q8WqFfeaY=biLkVcLq=JHqVepeea0=as0db9vqpepesP0xe9Fve9Fve9GapdbaqaaeGacaGaaiaabeqaamqadiabaaGcbaGafmOyaiMbaSaaaaa@3E4A@ just cancel c→
 MathType@MTEF@5@5@+=feaafiart1ev1aaatCvAUfKttLearuWrP9MDH5MBPbIqV92AaeXatLxBI9gBaebbnrfifHhDYfgasaacH8akY=wiFfYdH8Gipec8Eeeu0xXdbba9frFj0=OqFfea0dXdd9vqai=hGuQ8kuc9pgc9s8qqaq=dirpe0xb9q8qiLsFr0=vr0=vr0dc8meaabaqaciaacaGaaeqabaqabeGadaaakeaacuWGJbWygaWcaaaa@2E0D@ and – (a→
 MathType@MTEF@5@5@+=feaafiart1ev1aaatCvAUfKttLearuWrP9MDH5MBPbIqV92AaeXatLxBI9gBamXvP5wqSXMqHnxAJn0BKvguHDwzZbqegyvzYrwyUfgarqqtubsr4rNCHbGeaGqiA8vkIkVAFgIELiFeLkFeLk=iY=Hhbbf9v8qqaqFr0xc9pk0xbba9q8WqFfeaY=biLkVcLq=JHqVepeea0=as0db9vqpepesP0xe9Fve9Fve9GapdbaqaaeGacaGaaiaabeqaamqadiabaaGcbaGafmyyaeMbaSaaaaa@3E48@ + b→
 MathType@MTEF@5@5@+=feaafiart1ev1aaatCvAUfKttLearuWrP9MDH5MBPbIqV92AaeXatLxBI9gBamXvP5wqSXMqHnxAJn0BKvguHDwzZbqegyvzYrwyUfgarqqtubsr4rNCHbGeaGqiA8vkIkVAFgIELiFeLkFeLk=iY=Hhbbf9v8qqaqFr0xc9pk0xbba9q8WqFfeaY=biLkVcLq=JHqVepeea0=as0db9vqpepesP0xe9Fve9Fve9GapdbaqaaeGacaGaaiaabeqaamqadiabaaGcbaGafmOyaiMbaSaaaaa@3E4A@) will provide the same pseudo current density map as well!

In addition, if curl B→
 MathType@MTEF@5@5@+=feaafiart1ev1aaatCvAUfKttLearuWrP9MDH5MBPbIqV92AaeXatLxBI9gBamXvP5wqSXMqHnxAJn0BKvguHDwzZbqegyvzYrwyUfgarqqtubsr4rNCHbGeaGqiA8vkIkVAFgIELiFeLkFeLk=iY=Hhbbf9v8qqaqFr0xc9pk0xbba9q8WqFfeaY=biLkVcLq=JHqVepeea0=as0db9vqpepesP0xe9Fve9Fve9GapdbaqaaeGacaGaaiaabeqaamqadiabaaGcbaGafmOqaiKbaSaaaaa@3E0A@ = 0 then

∂Bz∂y=∂By∂z; ∂Bx∂z=∂Bz∂x; ∂By∂x=∂Bx∂y     (19)
 MathType@MTEF@5@5@+=feaafiart1ev1aaatCvAUfKttLearuWrP9MDH5MBPbIqV92AaeXatLxBI9gBaebbnrfifHhDYfgasaacH8akY=wiFfYdH8Gipec8Eeeu0xXdbba9frFj0=OqFfea0dXdd9vqai=hGuQ8kuc9pgc9s8qqaq=dirpe0xb9q8qiLsFr0=vr0=vr0dc8meaabaqaciaacaGaaeqabaqabeGadaaakeaadaWcaaqaaiabgkGi2kabdkeacnaaBaaaleaacqWG6bGEaeqaaaGcbaGaeyOaIyRaemyEaKhaaiabg2da9maalaaabaGaeyOaIyRaemOqai0aaSbaaSqaaiabdMha5bqabaaakeaacqGHciITcqWG6bGEaaGaei4oaSJaeeiiaaYaaSaaaeaacqGHciITcqWGcbGqdaWgaaWcbaGaemiEaGhabeaaaOqaaiabgkGi2kabdQha6baacqGH9aqpdaWcaaqaaiabgkGi2kabdkeacnaaBaaaleaacqWG6bGEaeqaaaGcbaGaeyOaIyRaemiEaGhaaiabcUda7iabbccaGmaalaaabaGaeyOaIyRaemOqai0aaSbaaSqaaiabdMha5bqabaaakeaacqGHciITcqWG4baEaaGaeyypa0ZaaSaaaeaacqGHciITcqWGcbGqdaWgaaWcbaGaemiEaGhabeaaaOqaaiabgkGi2kabdMha5baacaWLjaGaaCzcamaabmaabaGaeGymaeJaeGyoaKdacaGLOaGaayzkaaaaaa@6283@

and only 2 components *B*_*x*_, *B*_*y*_, or *B*_*x*_, *B*_*z*_, or *B*_*y*_, *B*_*z *_are necessary to construct a→
 MathType@MTEF@5@5@+=feaafiart1ev1aaatCvAUfKttLearuWrP9MDH5MBPbIqV92AaeXatLxBI9gBamXvP5wqSXMqHnxAJn0BKvguHDwzZbqegyvzYrwyUfgarqqtubsr4rNCHbGeaGqiA8vkIkVAFgIELiFeLkFeLk=iY=Hhbbf9v8qqaqFr0xc9pk0xbba9q8WqFfeaY=biLkVcLq=JHqVepeea0=as0db9vqpepesP0xe9Fve9Fve9GapdbaqaaeGacaGaaiaabeqaamqadiabaaGcbaGafmyyaeMbaSaaaaa@3E48@, b→
 MathType@MTEF@5@5@+=feaafiart1ev1aaatCvAUfKttLearuWrP9MDH5MBPbIqV92AaeXatLxBI9gBamXvP5wqSXMqHnxAJn0BKvguHDwzZbqegyvzYrwyUfgarqqtubsr4rNCHbGeaGqiA8vkIkVAFgIELiFeLkFeLk=iY=Hhbbf9v8qqaqFr0xc9pk0xbba9q8WqFfeaY=biLkVcLq=JHqVepeea0=as0db9vqpepesP0xe9Fve9Fve9GapdbaqaaeGacaGaaiaabeqaamqadiabaaGcbaGafmOyaiMbaSaaaaa@3E4A@, and c→
 MathType@MTEF@5@5@+=feaafiart1ev1aaatCvAUfKttLearuWrP9MDH5MBPbIqV92AaeXatLxBI9gBaebbnrfifHhDYfgasaacH8akY=wiFfYdH8Gipec8Eeeu0xXdbba9frFj0=OqFfea0dXdd9vqai=hGuQ8kuc9pgc9s8qqaq=dirpe0xb9q8qiLsFr0=vr0=vr0dc8meaabaqaciaacaGaaeqabaqabeGadaaakeaacuWGJbWygaWcaaaa@2E0D@.

For example, by exploiting relations (19) the three vectors constructed with *B*_*x*_, *B*_*y *_yield

a→=∂Bx∂ze→y−∂Bx∂ye→z=∂Bx∂ze→y−∂By∂xe→z,     (20)
 MathType@MTEF@5@5@+=feaafiart1ev1aaatCvAUfKttLearuWrP9MDH5MBPbIqV92AaeXatLxBI9gBaebbnrfifHhDYfgasaacH8akY=wiFfYdH8Gipec8Eeeu0xXdbba9frFj0=OqFfea0dXdd9vqai=hGuQ8kuc9pgc9s8qqaq=dirpe0xb9q8qiLsFr0=vr0=vr0dc8meaabaqaciaacaGaaeqabaqabeGadaaakeaacuWGHbqygaWcaiabg2da9maalaaabaGaeyOaIyRaemOqai0aaSbaaSqaaiabdIha4bqabaaakeaacqGHciITcqWG6bGEaaGafmyzauMbaSaadaWgaaWcbaGaemyEaKhabeaakiabgkHiTmaalaaabaGaeyOaIyRaemOqai0aaSbaaSqaaiabdIha4bqabaaakeaacqGHciITcqWG5bqEaaGafmyzauMbaSaadaWgaaWcbaGaemOEaOhabeaakiabg2da9maalaaabaGaeyOaIyRaemOqai0aaSbaaSqaaiabdIha4bqabaaakeaacqGHciITcqWG6bGEaaGafmyzauMbaSaadaWgaaWcbaGaemyEaKhabeaakiabgkHiTmaalaaabaGaeyOaIyRaemOqai0aaSbaaSqaaiabdMha5bqabaaakeaacqGHciITcqWG4baEaaGafmyzauMbaSaadaWgaaWcbaGaemOEaOhabeaakiabcYcaSiaaxMaacaWLjaWaaeWaaeaacqaIYaGmcqaIWaamaiaawIcacaGLPaaaaaa@6028@

b→=∂By∂xe→z−∂By∂ze→x=∂Bx∂ye→z−∂By∂ze→x,     (21)
 MathType@MTEF@5@5@+=feaafiart1ev1aaatCvAUfKttLearuWrP9MDH5MBPbIqV92AaeXatLxBI9gBaebbnrfifHhDYfgasaacH8akY=wiFfYdH8Gipec8Eeeu0xXdbba9frFj0=OqFfea0dXdd9vqai=hGuQ8kuc9pgc9s8qqaq=dirpe0xb9q8qiLsFr0=vr0=vr0dc8meaabaqaciaacaGaaeqabaqabeGadaaakeaacuWGIbGygaWcaiabg2da9maalaaabaGaeyOaIyRaemOqai0aaSbaaSqaaiabdMha5bqabaaakeaacqGHciITcqWG4baEaaGafmyzauMbaSaadaWgaaWcbaGaemOEaOhabeaakiabgkHiTmaalaaabaGaeyOaIyRaemOqai0aaSbaaSqaaiabdMha5bqabaaakeaacqGHciITcqWG6bGEaaGafmyzauMbaSaadaWgaaWcbaGaemiEaGhabeaakiabg2da9maalaaabaGaeyOaIyRaemOqai0aaSbaaSqaaiabdIha4bqabaaakeaacqGHciITcqWG5bqEaaGafmyzauMbaSaadaWgaaWcbaGaemOEaOhabeaakiabgkHiTmaalaaabaGaeyOaIyRaemOqai0aaSbaaSqaaiabdMha5bqabaaakeaacqGHciITcqWG6bGEaaGafmyzauMbaSaadaWgaaWcbaGaemiEaGhabeaakiabcYcaSiaaxMaacaWLjaWaaeWaaeaacqaIYaGmcqaIXaqmaiaawIcacaGLPaaaaaa@602C@

c→=∂By∂ze→x−∂Bx∂ze→y.     (22)
 MathType@MTEF@5@5@+=feaafiart1ev1aaatCvAUfKttLearuWrP9MDH5MBPbIqV92AaeXatLxBI9gBaebbnrfifHhDYfgasaacH8akY=wiFfYdH8Gipec8Eeeu0xXdbba9frFj0=OqFfea0dXdd9vqai=hGuQ8kuc9pgc9s8qqaq=dirpe0xb9q8qiLsFr0=vr0=vr0dc8meaabaqaciaacaGaaeqabaqabeGadaaakeaacuWGJbWygaWcaiabg2da9maalaaabaGaeyOaIyRaemOqai0aaSbaaSqaaiabdMha5bqabaaakeaacqGHciITcqWG6bGEaaGafmyzauMbaSaadaWgaaWcbaGaemiEaGhabeaakiabgkHiTmaalaaabaGaeyOaIyRaemOqai0aaSbaaSqaaiabdIha4bqabaaakeaacqGHciITcqWG6bGEaaGafmyzauMbaSaadaWgaaWcbaGaemyEaKhabeaakiabc6caUiaaxMaacaWLjaWaaeWaaeaacqaIYaGmcqaIYaGmaiaawIcacaGLPaaaaaa@49EB@

**Figure 10:**
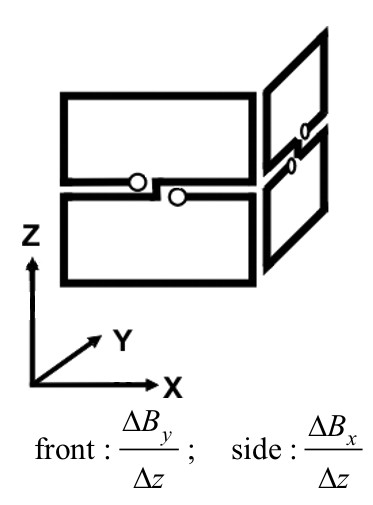
Planar vertical gradiometers as a hardware realization for performing direct HC-transformations and the related difference quotients.

The last relation for c→
 MathType@MTEF@5@5@+=feaafiart1ev1aaatCvAUfKttLearuWrP9MDH5MBPbIqV92AaeXatLxBI9gBaebbnrfifHhDYfgasaacH8akY=wiFfYdH8Gipec8Eeeu0xXdbba9frFj0=OqFfea0dXdd9vqai=hGuQ8kuc9pgc9s8qqaq=dirpe0xb9q8qiLsFr0=vr0=vr0dc8meaabaqaciaacaGaaeqabaqabeGadaaakeaacuWGJbWygaWcaaaa@2E0D@ may be easily realized by another SQUID-system hardware consisting of vertically oriented planar gradiometers [34]. This system approximates the partial derivative of *B*_*y *_and *B*_*x *_with respect to *z*. Thus also with that system a direct acquisition of the pseudo current density map is possible (cf. Fig. 10).

Finally, the newer vectormagnetometer systems [35,36] also allow an appropriate combination of partial derivatives leading to

c→=12(∂Bz∂ye→x−∂Bz∂xe→y+∂By∂ze→x−∂Bx∂ze→y).     (23)
 MathType@MTEF@5@5@+=feaafiart1ev1aaatCvAUfKttLearuWrP9MDH5MBPbIqV92AaeXatLxBI9gBaebbnrfifHhDYfgasaacH8akY=wiFfYdH8Gipec8Eeeu0xXdbba9frFj0=OqFfea0dXdd9vqai=hGuQ8kuc9pgc9s8qqaq=dirpe0xb9q8qiLsFr0=vr0=vr0dc8meaabaqaciaacaGaaeqabaqabeGadaaakeaacuWGJbWygaWcaiabg2da9maalaaabaGaeGymaedabaGaeGOmaidaamaabmaabaWaaSaaaeaacqGHciITcqWGcbGqdaWgaaWcbaGaemOEaOhabeaaaOqaaiabgkGi2kabdMha5baacuWGLbqzgaWcamaaBaaaleaacqWG4baEaeqaaOGaeyOeI0YaaSaaaeaacqGHciITcqWGcbGqdaWgaaWcbaGaemOEaOhabeaaaOqaaiabgkGi2kabdIha4baacuWGLbqzgaWcamaaBaaaleaacqWG5bqEaeqaaOGaey4kaSYaaSaaaeaacqGHciITcqWGcbGqdaWgaaWcbaGaemyEaKhabeaaaOqaaiabgkGi2kabdQha6baacuWGLbqzgaWcamaaBaaaleaacqWG4baEaeqaaOGaeyOeI0YaaSaaaeaacqGHciITcqWGcbGqdaWgaaWcbaGaemiEaGhabeaaaOqaaiabgkGi2kabdQha6baacuWGLbqzgaWcamaaBaaaleaacqWG5bqEaeqaaaGccaGLOaGaayzkaaGaeiOla4IaaCzcaiaaxMaadaqadaqaaiabikdaYiabiodaZaGaayjkaiaawMcaaaaa@638D@

Again, the same pseudo current density map occurs, but the signal to noise ratio will be enhanced, as all three vector components of B→
 MathType@MTEF@5@5@+=feaafiart1ev1aaatCvAUfKttLearuWrP9MDH5MBPbIqV92AaeXatLxBI9gBamXvP5wqSXMqHnxAJn0BKvguHDwzZbqegyvzYrwyUfgarqqtubsr4rNCHbGeaGqiA8vkIkVAFgIELiFeLkFeLk=iY=Hhbbf9v8qqaqFr0xc9pk0xbba9q8WqFfeaY=biLkVcLq=JHqVepeea0=as0db9vqpepesP0xe9Fve9Fve9GapdbaqaaeGacaGaaiaabeqaamqadiabaaGcbaGafmOqaiKbaSaaaaa@3E0A@(r→
 MathType@MTEF@5@5@+=feaafiart1ev1aaatCvAUfKttLearuWrP9MDH5MBPbIqV92AaeXatLxBI9gBamXvP5wqSXMqHnxAJn0BKvguHDwzZbqegyvzYrwyUfgarqqtubsr4rNCHbGeaGqiA8vkIkVAFgIELiFeLkFeLk=iY=Hhbbf9v8qqaqFr0xc9pk0xbba9q8WqFfeaY=biLkVcLq=JHqVepeea0=as0db9vqpepesP0xe9Fve9Fve9GapdbaqaaeGacaGaaiaabeqaamqadiabaaGcbaGafmOCaiNbaSaaaaa@3E6A@) are utilized.

### Visualizing dynamics by creating a sequence of pseudo current density maps for MCG data

The perception of dynamic phenomena is considerably enhanced by viewing movies.

**Figure 11:**
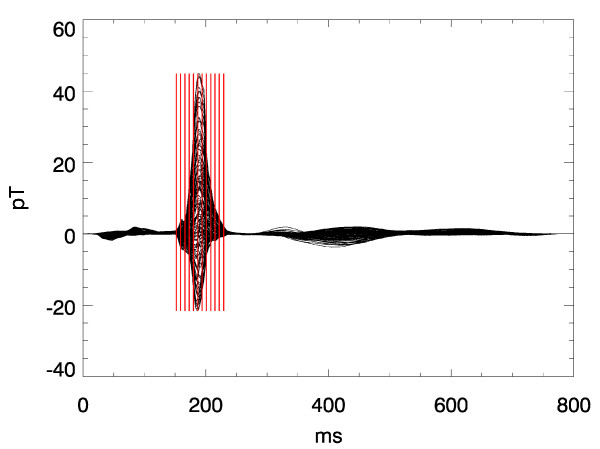
Butterfly plot of a multichannel magnetocardiogram. The cursors indicate the time instants of the frames in Fig. 12.

**Figure 12:**
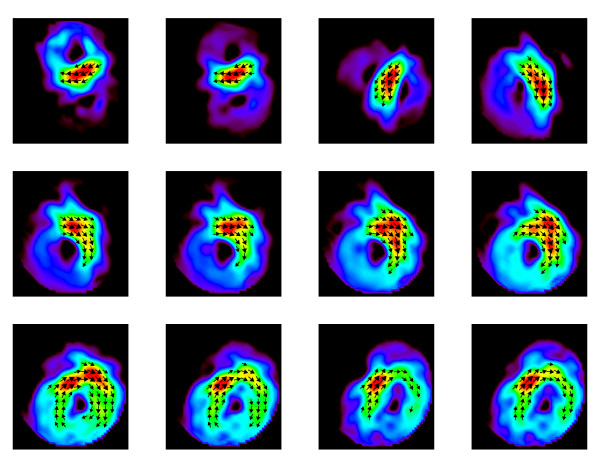
Frames of a video-sequence of pseudo current density maps during the QRS-complex.

A sequence of frames might give an impression of what can be expressed by a movie clip. Figs. 12 displays such a sequence of frames showing the evolution of PCD-maps gained from the multichannel MCG during a heart beat of a healthy volunteer. Due to the higher dynamics during the QRS-complex the frame rate is higher there than during the ST-phase. The start of the activation sequence in the septum, the downwards propagation to the apex, and the following depolarization (Figs. 11 and 12) are visible as it is expected from textbook knowledge. The corresponding movie is attached as an additional data file (see Additional file 1).

**Figure 14:**
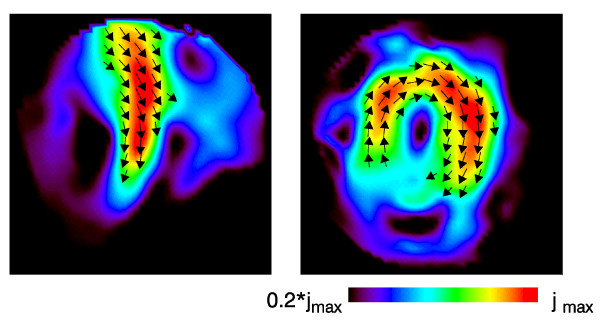
Frames of a video-sequence of pseudo current density maps during the T- and U-wave.

It is obvious that a PCD-map at the end of the T-wave may serve more consistently for evaluating dispersion of repolarization (Fig. 14) than a *B*_*z*_-map with its zero-isofield-line.

**Figure 13:**
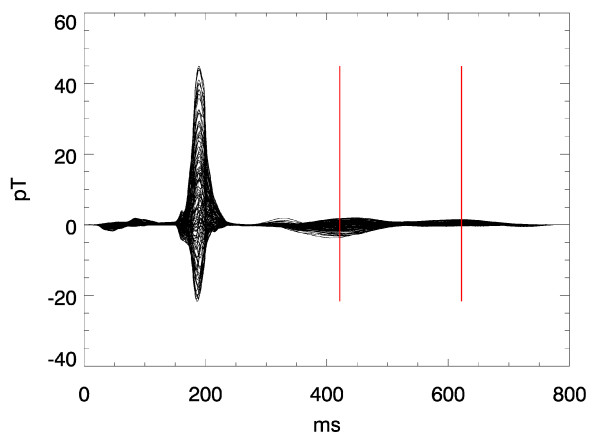
Butterfly plot of a multichannel magnetocardiogram. The cursors indicate the time instants of the frames in Fig. 14.

Another interesting aspect is the difference in spatial field configuration between end of the T-wave and the U-wave. The real nature of the U-wave is still under debate. But any hypothesis should consider the fact shown here (and confirmed in many other cases) that the spatial origin of the excitation that generates the ECG or MCG at the end of the T-wave differs markedly from that of the U-wave (Figs. 13 and 14).

## Conclusion

In this work we presented examples of electrophysiological measurements where the use of PCD-maps is meaningful. PCD-maps allow in these selected cases an estimate of the underlying currents and also of the temporal behavior of the current propagation. On the other hand, the PCD-maps are only a 2D-presentation of a 3D-current distribution and may deviate considerable from the real current distribution.

We described the analytical basis of PCD-maps and showed that there exist alternative PCD-map presentations if other field components then *B*_*z *_are also taken into account. Additionally we extended the PCD-map method to spherical coordinates as used in MEG.

PCD-maps are very interesting nowadays due to hardware realizations by special designed coil configurations or vector magnetometers. Vector magnetometry allows the recording of all magnetic field components and thus the direct realization of all proposed PCD-map cases.

The advantages of pseudo current density maps besides their intuitive character ("maximum signal is where the action is") are their model- and hardware-independence. While sophisticated inverse methods and filter techniques (e.g. the synthetic aperture beamformer [37]) may lead to more exact results with respect to the real current density distribution, they are hard to validate and require advanced data processing. In multicentric clinical studies, where comparability of measurement results between different groups is a key issue, PCD-maps might serve as a basis to exchange results. PCD-maps from such different SQUID-systems as those with planar horizontal, planar vertical magnetometers or gradiometers, or vectormagnetometers differ only slightly and are still traceable back to the original measurement results (up to an additive constant).

## Competing interests

The author(s) declare that they have no competing interests.

## Authors' contributions

WH: theory, mathematics

US: critical revision and supportive contributions

MB: critical revision, chapter on MNG and MEG recordings

OK: critical revision, results of MCG-investigations

AM: clinical investigation and support

HK: corresponding author, drafting of the manuscript, visualization, animation, and final approval

## Supplementary Material

Additional File 1 MCG-movies. The attached Power Point file "MCG-movies.ppt" contains movies (animated GIF; runs only in recent Power Point versions) of the map sequences partly shown in figures 2, 3, and 13. Click here for file
