# Supplementary material for: Pseudo current density maps of electrophysiological heart, nerve or brain function and their physical basis
Source: Biomagn Res Technol. 2006 Oct 13;4:5. doi: 10.1186/1477-044X-4-5 (PMC1660567; doi:10.1186/1477-044X-4-5)
Supplement: Additional File 1 — MCG-movies. The attached Power Point file "MCG-movies.ppt" contains movies (animated GIF; runs only in recent Power Point versions) of the map sequences partly shown in figures 2, 3, and 13. [file 1477-044X-4-5-S1.ppt]

## Slide 1
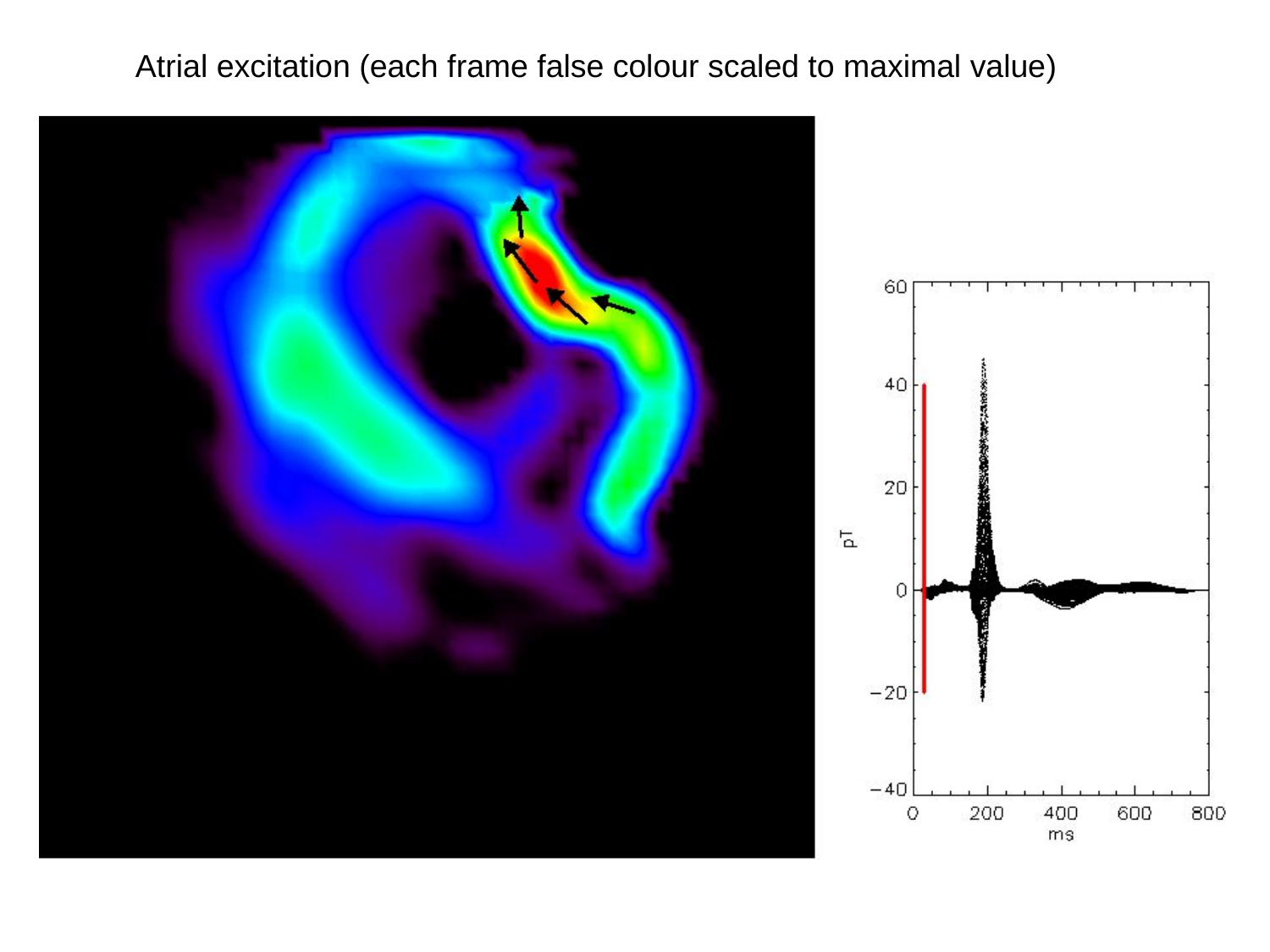

Atrial excitation (each frame false colour scaled to maximal value)

## Slide 2
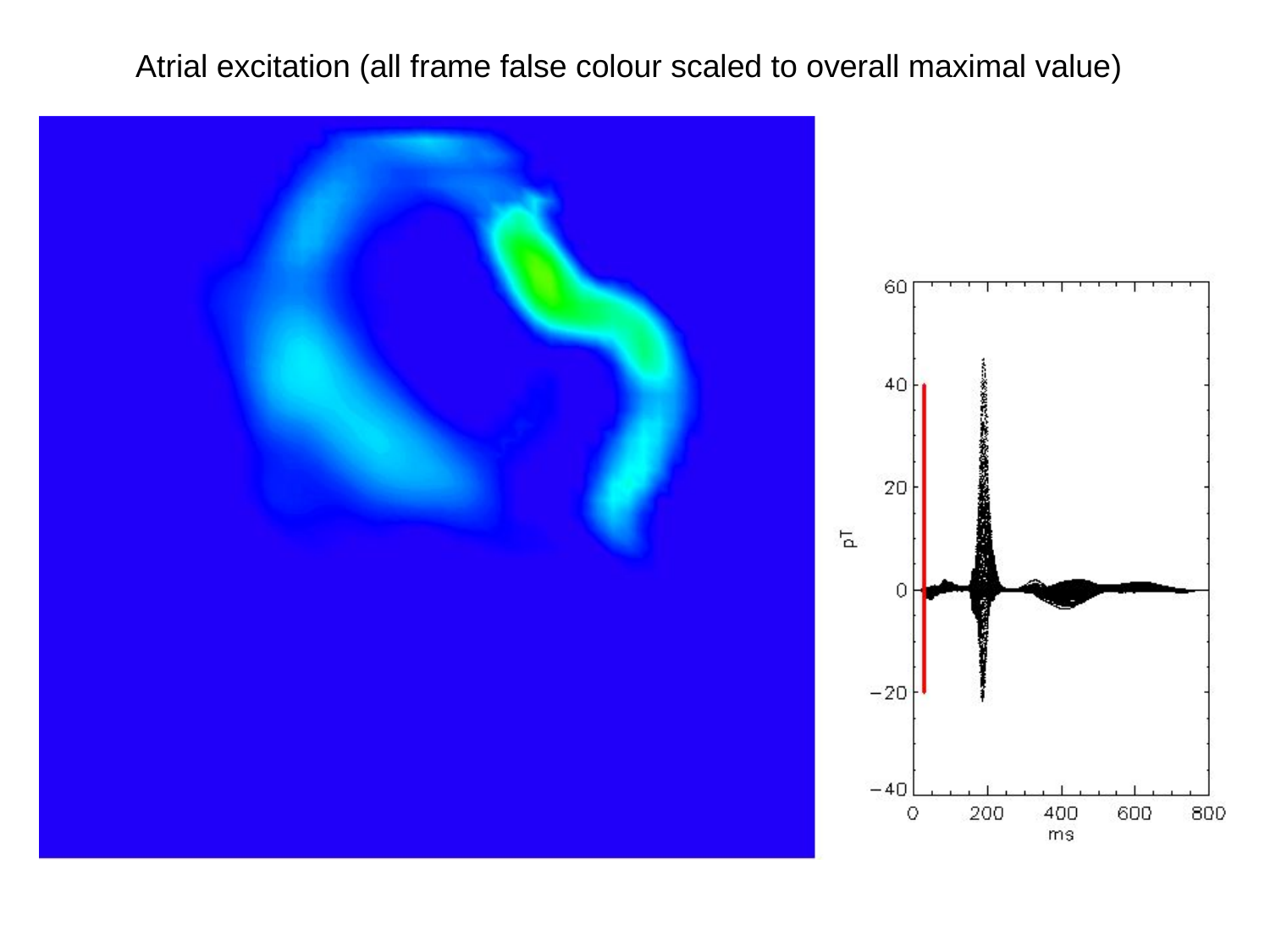

Atrial excitation (all frame false colour scaled to overall maximal value)

## Slide 3
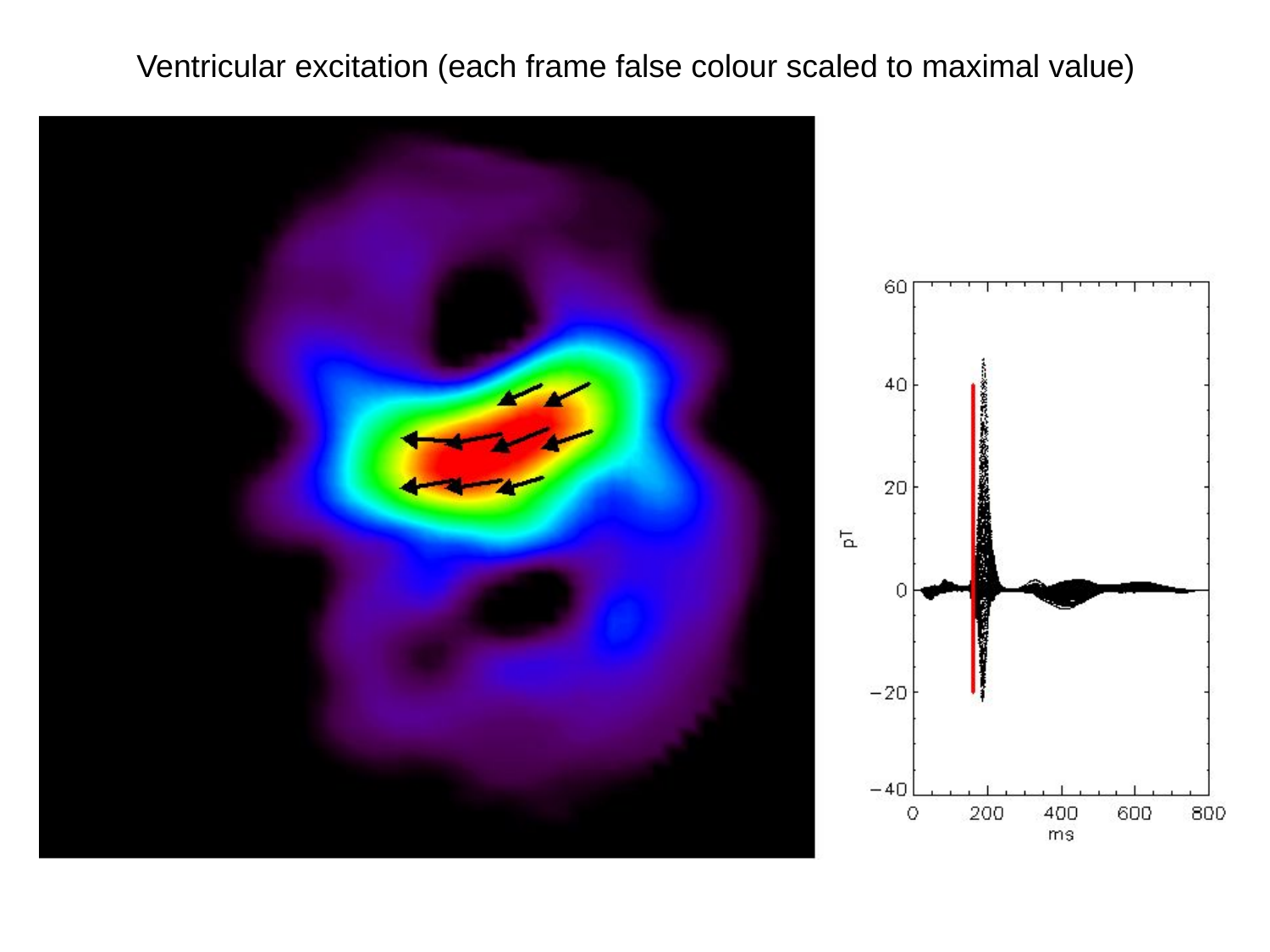

Ventricular excitation (each frame false colour scaled to maximal value)
